# Efficiency and Complication of 577-nm Laser Membranotomy for the Treatment of Retinal Sub-Inner Limiting Membrane Hemorrhage

**DOI:** 10.3389/fopht.2022.935188

**Published:** 2022-07-13

**Authors:** Yongpeng Zhang, Jipeng Li, Xusheng Cao, Haiying Zhou, Liyun Jia, Liqin Gao, Zhihua Li, Xifang Zhang, Haicheng She, Kai Ma, Xiaoyan Peng

**Affiliations:** Beijing Tongren Eye Center, Beijing Tongren Hospital, Capital Medical University, Beijing Key Laboratory of Ophthalmology and Visual Science, Beijing, China

**Keywords:** inner limiting membrane, laser, OCT, complication, hemorrhage, 577nm

## Abstract

**Objective:**

We investigated the clinical efficiency and complications of treatment of retinal sub-inner limiting membrane (sub-ILM) hemorrhage by 577-nm semiconductive laser membranotomy.

**Methods:**

The clinical features, ocular fundus photography, and SD-OCT image of patients who received 577-nm laser membranotomy for sub-ILM hemorrhage were assessed from January 2017 to April 2022 in this retrospective case-series study.

**Results:**

A total of 19 patients (19 eyes) were treated for sub-ILM hemorrhage of the macula, in which eight were women and 11 were men. The age was 15–83 years (with an average age of 49.05 ± 19.41 years old). The right eye was affected in 12 patients, the left eye in seven patients. The follow-up period after laser treatment was from 0.5 to 9 months (with an average follow-up time of 3.25 ± 2.45 months). Treatment was not successful in one patient, and 577-nm laser membranotomy was successful in 18 patients (equaling a success rate of 94.74%). The best corrected visual acuity (BCVA) before laser treatment was from figure count to 40/200, and the BCVA after laser treatment was from 20/2000 to 20/20. Complications after laser treatment comprised macular hole (one patient), macular epi-membrane (one patient), vitreous hemorrhage without absorption (two patients), and sub-ILM cavity (12 patients).

**Conclusions:**

The 577-nm laser is effective in treating sub-ILM hemorrhage and has a high success rate. Posttreatment complications should be monitored, and vitrectomy was needed with long-lasting vitreous hemorrhage and macular hole.

## Introduction

Sub-inner limiting membrane (sub-ILM) hemorrhage is seldom seen in clinic but is a particularly concerning retinal condition because it can cause sudden and severe vision loss. Spontaneous resorption of the blood entrapped in the sub-ILM space tends to be slow and may result in irreversible retinal damage and long-standing visual impairment (1–5).

Current treatment methods for sub-ILM hemorrhage include observation, membranotomy by laser, intravitreal gas injection, intravitreal injection of t-PA, intravitreal injection of anti-VEGF, and vitrectomy with ILM peeling. Sub-ILM hemorrhage may have toxic effects on the retina, including a macular hole that may be concealed under the hemorrhage; thus, early intervention is key to improved prognosis.

577-nm semiconductor lasers are widely used in the treatment of fundus diseases, primarily due to its stable power output and ability to accurately aim light. These lasers can also be used for the treatment of sub-ILM hemorrhage by burst effect. In addition, 577-nm semiconductor lasers are better than YAG lasers in treating comorbid ocular fundus diseases simultaneously, including diabetic retinopathy (DR) and retinal arterial microaneurysm (RAM).

As the incidence of sub-ILM hemorrhage is fairly low and the existing research primarily consists of case reports rather than in-depth controlled research studies, we have limited knowledge about the clinical efficiency, safety, and complications of laser membranotomy for sub-ILM hemorrhage. Here we present 19 cases of sub-ILM hemorrhage treated by a 577-nm semiconductor laser and assess the image features and clinical efficiency and complications after laser treatment.

## Methods

### Ethical Approval

Ethics approval was obtained from the Institutional Ethics Committee of Beijing Tongren Hospital, Capital Medical University. The study was performed in accordance with the Declaration of Helsinki. Written informed consent was obtained from all examined individuals before they underwent laser treatment. In cases in which the patient was younger than 18 years of age, consent was obtained from a parent or legal guardian.

### Study Population and Treatment Procedures

This was an observational, retrospective case-series study. Nineteen patients (19 eyes) of sub-ILM hemorrhage from Tongren Eye Center, Capital Medical University, were analyzed in this retrospective case-series study of patients undergoing 577-nm semiconductor laser treatment at our institution from January 2017 to April 2022.

A comprehensive ophthalmologic examination, including best corrected visual acuity (BCVA), slit-lamp examination, intraocular pressure, ocular fundus examination with biomicroscopy, and indirect ophthalmoscopy, were performed preoperatively as well as postoperatively.

Fundus photography (Canon™ or Optos™) and SD-OCT (Heidelberg™ or Optovue™) were taken for every patient before and after laser treatment.

### Laser Membranotomy

The laser wavelength applied in this study was 577 nm (Lumenis™ Vision One). The laser power was from 300 to 500 mW. The laser spot size was 200 μm. The laser’s exposure time was 200 ms. The number of laser spots was from 1 to 14. The laser membranotomy location was inferior of the sub-ILM hemorrhage and about 200 μm (the size of a laser spot) from the hemorrhage edge to avoid damage to normal retinal tissue.

## Results

A total of 19 patients (19 eyes) were eligible for analyses after 577-nm membranotomy of sub-ILM hemorrhage. Eight patients were women, and 11 patients were men (age range: 15–83 years old; mean age: 49.05 ± 19.41 years old). The right eye was affected in 12 patients, and the left eye was affected in seven patients The causes of sub-ILM hemorrhage were Valsalva retinopathy (10 cases), RAM (five cases), DR (three cases), and ocular blunt trauma (one case).

The time of symptom onset to laser intervention was 1–60 days (mean: 16.79 days). Drainage of sub-ILM hemorrhage was successful in 18 patients after laser membranotomy and failed in one patient; this latter patient was an outlier in terms of time between onset and intervention, with a duration of 60 days. Drainage could be seen immediately after laser burst in 13 cases and 1 day later in the other five successful cases. The absorption time of vitreous hemorrhage in the periphery could last several months, and we observed this absorption without oral medicine. We followed patients for 0.5–9 months (average: 3.25 ± 2.45 months) after laser treatment (**Table 1**).

**Table 1:** Demographic and clinical data of the 19 included patients.

Case no.	Gender	Diagnosis	Age	Symptom to laser interval (day)	Size of hemorrhage(DD)	Power of laser(mv)	Follow-uptime (month)	Initial BCVA	Final BCVA
1	Female	Valsalva	53	8	4 × 4	300	3	20/2,000	20/400
2	Male	Valsalva	29	7	3 × 3	300	3.5	20/2,000	20/20
3	Male	Valsalva	22	12	5 × 5	300–400	9	20/1,000	20/25
4	Male	DR	52	60	2 × 4	300–400	3.5	20/2,000	20/2,000
5	Female	RAM	78	15	3 × 3	400	4	FC	20/2,000
6	Female	DR	56	25	4 × 4	320–340	2.5	FC	20/100
7	Female	Valsalva	55	30	3 × 4	300–400	1.25	20/200	20/100
8	Male	Valsalva	60	19	3 × 4	300–500	5	FC	20/20
9	Male	Valsalva	15	11	4 × 5	300	7	20/1,000	20/20
10	Male	RAM	58	1	4 × 3	320	8	20/1,000	20/25
11	Male	blunt trauma	27	30	4 × 5	400–440	3.5	20/200	20/20
12	Female	DR	54	21	5 × 3	400	3.5	FC	20/400
13	Male	Valsalva	31	6	6 × 6	400	1	FC	20/125
14	Female	RAM	83	11	3 × 3	400	3	FC	20/100
15	Male	Valsalva	44	15	5 × 5	300–400	1.5	FC	20/1,000
16	Female	RAM	79	19	2 × 3	300–380	1	20/2,000	20/1,000
17	Female	Valsalva	43	10	3 × 3	300–340	0.5	20/400	20/100
18	Male	Valsalva	27	4	5 × 5	300–320	0.5	20/1,000	20/25
19	Male	RAM	66	16	3 × 3	340–380	0.5	FC	20/2,000

Case 4 was failure in laser treatment and received vitrectomy 1 month after laser. Case 7 and case 8 had persistent vitreous opacity after successful laser, and they received vitrectomy 2–3 months after laser. DR, diabetic retinopathy; RAM, retinal artery microaneurysm; FC, finger count.

**Figure 1:**
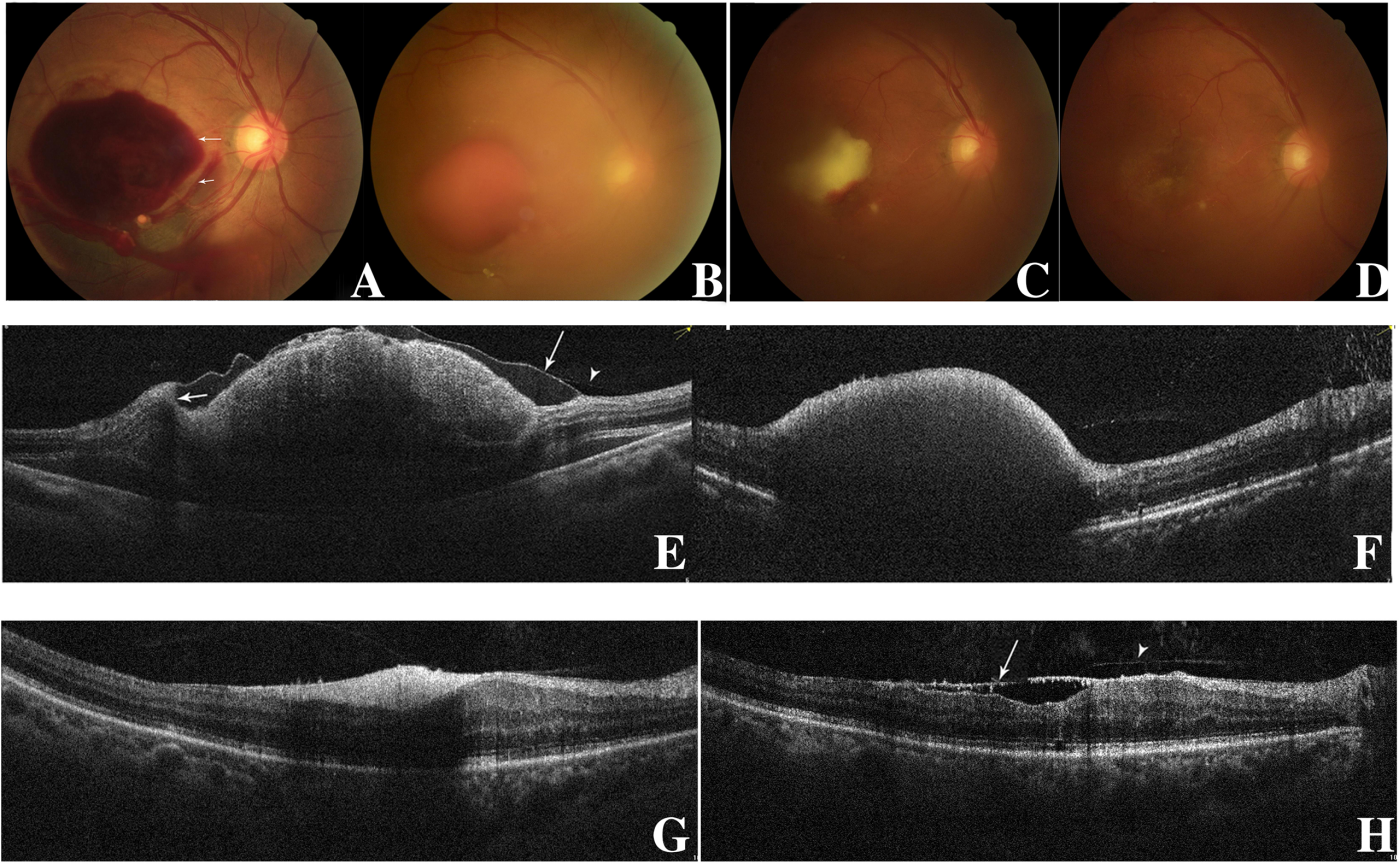
Fundus photography of the right eye (case 10) showing sub-ILM hemorrhage on the day of laser membranotomy. Blood could be seen flowing out from the ILM hole. There were two “rings” of hemorrhage which represented two kinds of sub-ILM hemorrhage as liquefying (short arrow) and coagulative (long arrow). This is different from the “double ring” sign, in which the “inner ring” is caused by the sub-ILM hemorrhage and the “outer ring” is caused by the sub-hyaloid hemorrhage. In the lower part, there was subretina hemorrhage **(A)**. At 1.5 months later, the sub-ILM hemorrhage became smaller but had not been absorbed completely **(B)**. Three months after laser treatment, the sub-ILM hemorrhage became even smaller but had not been absorbed completely. The color of sub-ILM hemorrhage changed from red to yellow-white **(C)**. Four months after laser treatment, the sub-ILM hemorrhage had been absorbed completely, and the BCVA recovered from 20/1,000 to 20/25 **(D)**. OCT image of the right eye on the day of laser. The posterior vitreous cortex (arrowhead) and the wavy-like ILM (long arrow) could be differentiated. The blood level of sub-ILM hemorrhage could be seen (short arrow) **(E)**. OCT image 1.5 months after laser treatment. The sub-ILM hemorrhage had not been absorbed completely, but the ILM had fallen back to the retina **(F)**. OCT image 3 months after laser treatment. The sub-ILM hemorrhage became smaller and denser but had not been absorbed completely **(G)**. OCT image 4 months after laser treatment showed that the sub-ILM hemorrhage had been absorbed completely and that the IIM had attached to the retina almost completely except for the fovea (arrow showing the ILM and arrowhead showing the posterior vitreous cortex) **(H)**.

**Figure 2:**
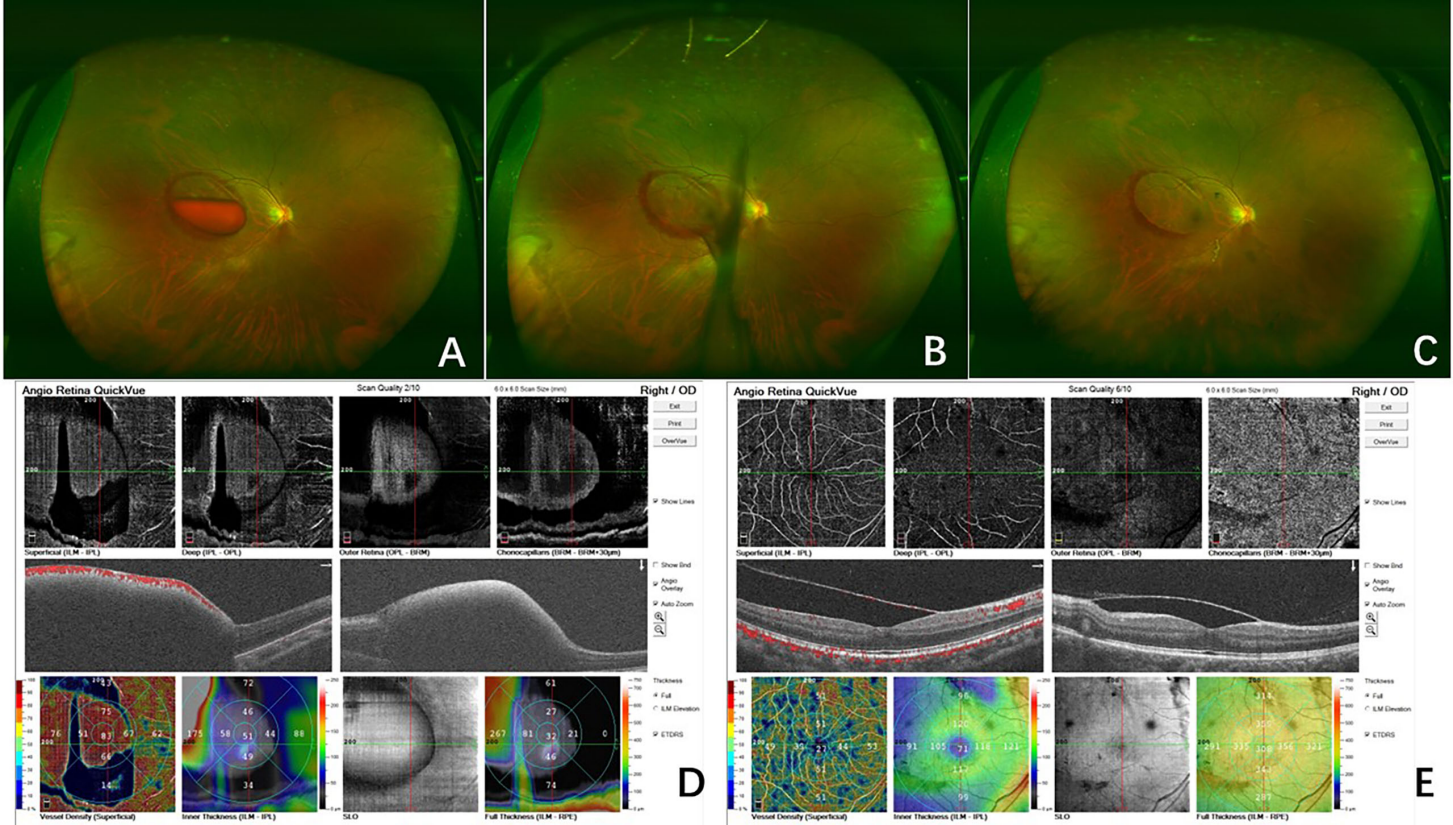
Fundus photography of the right eye (case 18) showed the sub-ILM hemorrhage with blood level before laser treatment **(A)**. On the day of the laser treatment, the sub-ILM hemorrhage was drained immediately, and the macular region was clear in 30 min **(B)**. At 5 days after laser treatment, the sub-ILM hemorrhage had been drained almost completely, and the BCVA recovered to 20/25 **(C)**. OCTA before laser treatment showed the sub-ILM hemorrhage **(D)**. In OCTA 5 days after laser treatment, the ILM was stiff and the sub-ILM cavity could be seen **(E)**.

**Figure 3:**
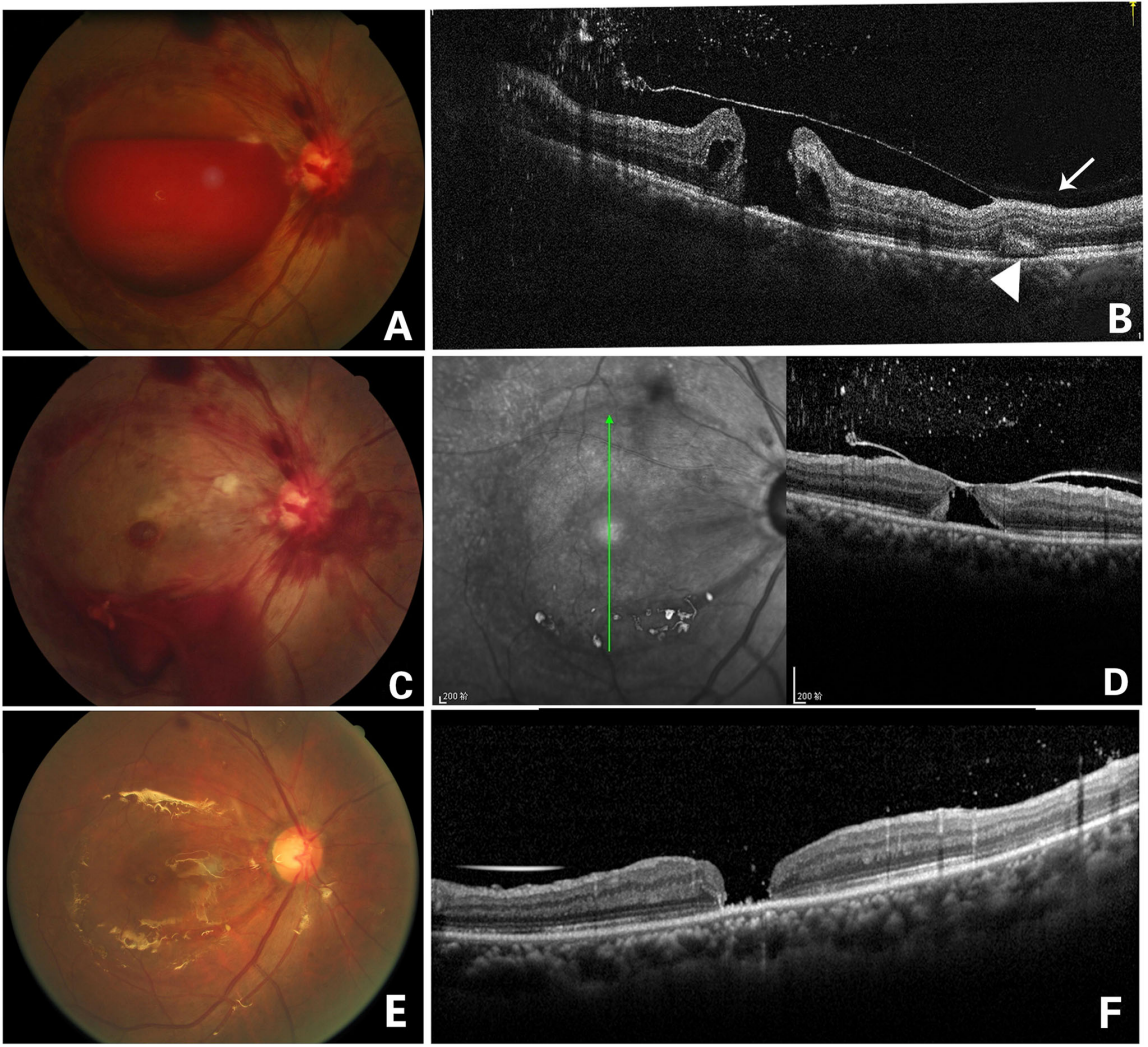
Fundus photography of the right eye (case 1) showing a large sub-ILM hemorrhage **(A)**. OCT image of the right eye 4 days after laser treatment showed ILM and an ILM hole, as well as multiple punctate hyperreflexia in the vitreous cavity. A full-thickness macular hole under the ILM could be seen. The posterior vitreous cortex was in front of the ILM attached to the retina (arrow), and a “patchy-like” structure was located in the outer retina (arrowhead)**(B)**. Fundus photography on the day of laser treatment showing that blood flowed out from the hole of the ILM and that vitreous bleeding and a round lesion of macula could be seen, which was later confirmed as a being macular hole **(C)**. In an OCT image 2 weeks later, the ILM fell back and attached to the surface of the macular hole, but the macular hole did not close **(D)**. Fundus photography of the right eye 2 months after laser treatment showing that vitrectomy with ILM peeling and silicone oil filling were performed in the right eye **(E)**. OCT image 2 months later after vitrectomy showed that the macular hole was still not closed. The “patchy-like” structure located in the outer retina disappeared **(F)**.

The SD-OCT image features of sub-ILM hemorrhage after 577-nm laser membranotomy could be classified into three areas: the morphology of the (1) ILM (2), fovea, and (3) outer retina. The ILM fall back to the retina (four cases; **Figure 1**). The hole on the ILM could be unsealed, forming a cavity between the ILM and the retina (12 cases; **Figure 2**). The fovea might have a normal contour (16 cases), macular hole (MH) (one case; **Figure 1**), or epiretinal membrane (two cases). We also observed “peg-like” structures (12 cases) in the outer retina (**Table 2**; **Figure 3**).

**Table 2:** The SD-OCT characteristics of 14 patients after laser treatment.

Case no.	ILM morphology after laser		Macular contour after laser			Outer retina
	ILM falling back	ILM stiff and forming sub-ILM cavity	Normal fovea contour	Macular hole	Pre-macular membrane	“peg-like” structure
1	Yes			Yes		Yes
2		Yes	Yes			
3		Yes	Yes			
5	Yes		Yes			Yes
6		Yes			Yes	
9		Yes	Yes			Yes
10	Yes		Yes			Yes
11		Yes	Yes			Yes
12		Yes			Yes	
13		Yes	Yes			Yes
14	Yes		Yes			Yes
15		Yes	Yes			Yes
16		Yes	Yes			Yes
17		Yes	Yes			Yes
18		Yes	Yes			Yes
19		Yes	Yes			Yes

Laser treatment was not successful for case 4. Case 7 and case 8 had persistent vitreous opacity after successful laser treatment, and they received vitrectomy 2–3 months after laser treatment. The SD-OCT picture could not be obtained after laser treatment of three cases.

## Discussion

“Premacular or preretinal hemorrhage” and “subhyaloidal hemorrhage” are commonly used synonyms for subhyaloidal and sub-ILM hemorrhages. Hemorrhage beneath the ILM is located within the neuro-retina, and the anatomically correct description should thus be “sub-ILM hemorrhage” (4).

Sub-ILM hemorrhage has been associated with various causes, the most common being Valsalva retinopathy and Terson’s syndrome; other causes include blunt trauma, ruptured RAM, blood dyscrasias, proliferative diabatic retinopathy (PDR), age-related macular degeneration, shaken baby syndrome, Dengue maculopathy, and Weil’s disease (1, 3, 6–17). In our study, 10 cases were Valsalva retinopathy (52.63%), five cases were RAM(26.32%), three cases were DR (15.79%), and one case was ocular blunt trauma(5.26%). Blood in sub-ILM hemorrhage spontaneously clears significantly slower than that in sub-hyaloid hemorrhage, and sub-hyaloid hemorrhage can often be observed for spontaneous improvement. The catabolites in sub-ILM hemorrhage may lead to retinal toxicity and proliferative vitreoretinopathy (3–5). In short, patients with sub-ILM hemorrhage who received earlier intervention have better visual outcomes (5).

Differentiating between the two types of hemorrhages clinically is a challenge. Specific signs in fundus photography may be the clue to sub-ILM hemorrhage. Specifically, sub-ILM hemorrhage appears as sharply demarcated, in which round hemorrhages at the posterior pole and a glistening light reflex of ILM can often be observed (2, 3). Another sign is the “double ring,” in which an “inner ring” caused by the sub-ILM hemorrhage and an “outer ring” caused by the sub-hyaloid hemorrhage can be observed (13, 18, 19), but this may not be true in some cases (such as case 7 in our study). Some authors have hypothesized that “perimacular folds” may be seen with large sub-ILM hemorrhage (12) and that “Arcus retinalis” could be a novel clinical marker of sub-ILM hemorrhage (20). To date, the only method to confirm the presence of sub-ILM hemorrhage remains intraoperative staining of the membrane overlying the hemorrhage and pathological confirmation (10). OCT remains the non-invasive mainstay for accurate diagnosis (1–4, 10, 11, 20).

OCT is the most effective method for diagnosing sub-ILM hemorrhage prior to treatment. Immediately above the level of sedimented blood, it can demonstrate two distinct membranes: a highly reflective band immediately above the hemorrhage corresponding to the ILM and an overlying patchy membrane with low optical reflectivity consistent with the posterior hyaloid (3, 4), and in some cases vertical “peg-like” structures can be seen in the outer retina (20). The systemic demonstration of SD-OCT characteristics after laser treatment is insufficient. In general, ILM cannot be distinguished on OCT because of its thickness and anatomical characteristics. Foveal ILM has a thickness of approximately 100 nm, whereas the parafoveal ILM has a thickness of up to 3 μm (21). If there is coexistence of sub-ILM with sub-hyaloid hemorrhage or if the posterior vitreous detachment is visible, the diagnosis is relatively easy; otherwise, using OCT as a tool for diagnosing sub-ILM hemorrhage remains difficult (3–5).

The treatment methods of sub-ILM hemorrhage include observation (17, 20, 22); membranotomy by argon, krypton, or YAG laser (1, 3, 9, 11, 18, 20, 23, 24),; intravitreal gas injection (14); intravitreal injection of tissue plasminogen activator (t-PA) (8); intravitreal injection of anti-VEGF; and vitrectomy with ILM peeling (3, 5–8, 10, 13, 15, 16, 20, 25).

577-nm lasers are widely used in the laser treatment of fundus diseases. As a semiconductor laser, its output power is steady and its aiming light is accurate. Various parameters such as power, laser spot diameter, and exposure time can be controlled. It has good operability and is suitable for laser treatment of sub-ILM hemorrhage. In addition, it has considerable advantages over YAG laser for patients with PDR who need pan-retinal photocoagulation at the same time. Compared with other treatment methods, the 577-nm laser has the advantages of causing less damage, allowing for faster recovery, and being cost-effective. It is especially suitable for young people, who often hope to quickly recover visual acuity and to reduce the impact of vitreoretinal surgery on the retina and lens, and for older adults, who often cannot tolerate or refuse vitrectomy.

In our study, 18 of the 19 patients were successfully treated with laser membranotomy, equating a success rate of 94.74%. Drainage failure occurred in one patient with DR and with whom the laser time was 60 days after onset. After laser treatment, BCVA increased in 16 cases, accounting for 84.21% of the cases (**Table 1**).

**Figure 4:**
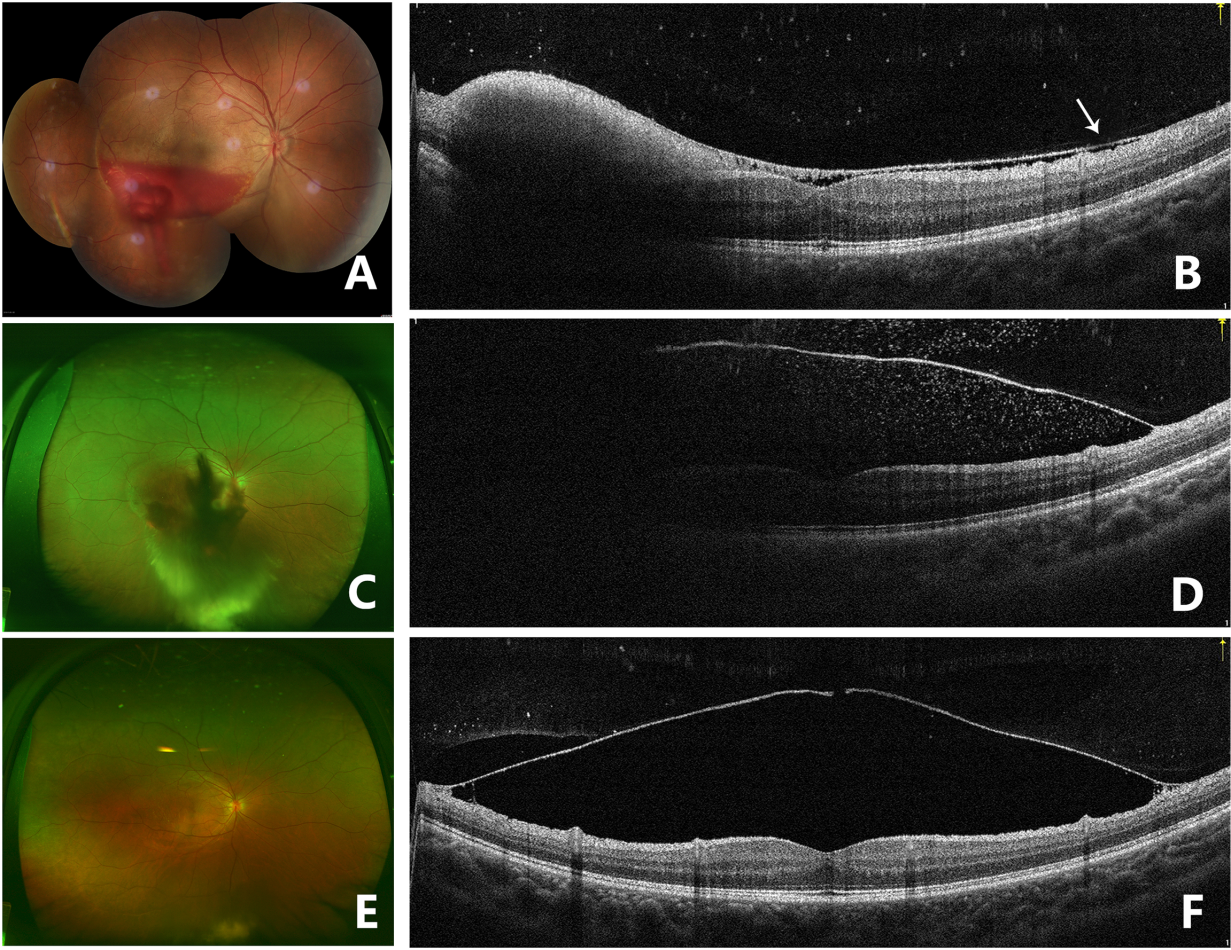
Fundus photography of the right eye (case 3) showing sub-ILM hemorrhage 5 days after laser membranotomy. Blood could be seen flowing out from the ILM hole **(A)**. OCT image on the 5th day after laser treatment in the right eye showed that the ILM above the macula dropped and that there was a thin hyporeflective band above the anterior surface of ILM, which was the posterior vitreous cortex (arrow). Hyperreflective spots could be seen on the retinal surface of the ILM, and residual hemorrhage under ILM could still be seen in the lower part of the fovea. The contour of the macula fovea was normal, but the surface of the retinal nerve fiber layer was undulant. A hyperreflective point could be seen in the vitreous cavity. The BCVA improved to 20/40 **(B)**. Fundus photography 1 month after laser treatment showed that sub-ILM hemorrhage and vitreous hemorrhage recurred and that the BCVA decreased to 20/400 **(C)**. OCT image of the right eye 1 month after laser treatment showed that the ILM was stiff and protruding and that there were hyperreflective points inside and outside the ILM **(D)**. Fundus photography showed that the sub-ILM hemorrhage had been absorbed 9 months after laser treatment, and vitreous opacity could be seen in the lower periphery of the vitreous cavity **(E)**. OCT image of the right eye 9 months after laser treatment showed that the ILM hole did not heal and that a cavity had formed below. Retinoschisis was seen at the attachment of the ILM to the retina **(F)**.

The case series included 13 cases of immediate drainage (defined as immediate hemorrhage drainage by laser) and five cases of delayed drainage (defined as hemorrhage drainage starting 1 day after laser treatment). It is relatively easy to drain hemorrhages an obvious fluid level immediately (such as with case 18, in which the sub-ILM hemorrhage had been drained almost completely 30 min after the laser treatment, **Figure 4**). However, hemorrhage without an obvious fluid level is much more difficult to drain. Age is a factor in drainage rate, as younger patients drained more quickly than older patients. Underlying causes of the hemorrhage are another reason for the drainage rate, as patients with Valsalva retinopathy drained more rapidly than patients with DR or RAM. Among the five patients with delayed drainage, three patients had diabetes mellitus and one was a 78-year-old woman. The complete drainage time of sub-ILM hemorrhage in the macular area after laser treatment lasted from 30 min to 4 months, and the average complete drainage time was 25.67 days. If the five patients with longer drainage time were excluded (two patients with 1 month, one patient with 1.5 months, one patient with 2 months, and one patient with 4 months), the average complete drainage time of the remaining 13 patients was 9 days.

Use of the 577-nm laser is an effective method to treat sub-ILM hemorrhage, but additional consideration is required to improve outcomes. First, the optimal time for laser treatment is no more than 21 days from the onset of hemorrhage (7, 9). In our case series, the time of onset to laser treatment varied from 1 to 60 days (with an average of 16.79 days). The size and thickness of the hemorrhage should also be taken into account, as hemorrhages that are too small or too thin should not be treated *via* laser. In our study, the size of the lesion was from 2 × 3 DD to 6 × 6 DD, which was safe for laser treatment. If the lower boundary of the sub-ILM hemorrhage is near the central area of the macula, the laser is not appropriate. If one laser spot is not successful, the laser burn should be changed to another position, and shooting the laser at the same point is not appropriate and could cause tissue damage. If the patient has frequent eye movement, the laser should be stopped in order to avoid damage to the normal retinal structure. The laser spot size that we used was 200 μm because drainage of the hemorrhage was difficult to accomplish through too small a hole. In contrast, a larger laser spot would disperse the energy, making it difficult to penetrate the ILM. Second, for cases with sub-ILM hemorrhage and PDR, laser membranotomy should be performed after photocoagulation in the lower peripheral retina, as this could lead to blood drainage that blocks the surgeon’s view.

The laser is safe and effective, but some complications should be considered. The complications of 577-nm treatment of sub-ILM hemorrhage include macular hole, macular epiretinal membrane, vitreous hemorrhage unabsorbed for an extended duration, and sub-ILM cavity formation.

The fovea might be a normal contour after laser treatment, but MH and epiretinal membrane have also been seen (2, 5, 7, 8, 13, 15, 22, 25). In this study, MH happened in one case, and epiretinal membrane occurred in two cases. Several causes may contribute to the formation of MH. It could be induced by hemorrhage breaking through the neurosensory layers, traction of ILM to the fovea, or toxicity of the long-lasting blood (22, 25). The primary closure rate of MH after sub-ILM hemorrhage seems to be low (57%) when compared with the rate for idiopathic MH (>90%) (14). In this study (**Figure 1**), the MH is unsealed even after vitrectomy and silicone oil tamponade. Although almost normal macular contour was achieved in most cases after laser treatment, BCVA of these cases were different (ranging from 20/2,000 to 20/20). The two patients (cases 5 and 12) who had poor vision prognosis had damage to the fovea because of RAM or DR rapture.

Even if the hemorrhage is drained into the vitreous cavity, the absorption is sometimes very slow, thus influencing the recovery of visual acuity and fundus observation (such as in cases 7 and 8). If the absorption of vitreous opacity time lasted longer than 1 or 2 months, vitrectomy should be applied.

The ILM is obviously thick in sub-ILM hemorrhage. The ILM is straight and stiff in most cases but can also be undulant (case 10) after laser treatment. The ILM can reattach to the retina or does not attach and instead forms a cavity between the ILM and the retina after membranotomy (**Figure 2**). In this study, the ILM fell back to the retina in four cases, and the time spent was from 2 weeks to 2 months. The ILM did not attach to the retina and had the “cavity-structure” in 12 cases in the research period. This phenomenon was not uncommon. Zhou et al. (9) noted several reasons for this complication, such as a large perforating puncture of the ILM and vitreous liquefaction (9). In our study, the average age of the ILM fallen back group was 68.00 years, and the average age of ILM not fallen back was 44.00 years.

Age seems to have an impact on posttreatment ILM position. ILM tends to fall back to the retina in older patients (with vitreous liquefaction) but sticks to the posterior hyaloid in younger patients (with much sticky vitreous and less liquefaction). We assessed the vitreous liquefaction before laser treatment and proved the vitreous liquefaction of all the patients who had ILM fallen back to the retina, so we hypothesized that vitreous liquefaction was the reason of ILM falling back instead of a “cavity-structure” formation. The sub-ILM cavity showed a “transparent plastic bag”-like appearance in the macular region, but it did not seem to affect the patient’s visual acuity, and the patients did not report obvious visual disturbance in our series. There were cases of patients with sub-ILM cavity after laser treatment, which did not resolve during the follow-up period. It is necessary to observe whether it will fall back with time and whether there is epiretinal membrane formation in an extended follow-up period.

This study had some limitations. Specifically, this study analyzed a limited number of cases (n = 19) and had a short follow-up period. In addition, this study did not enroll any healthy volunteers as control subjects, and no other laser-treatment or surgical options were assessed. Prospective and randomized trials with a larger sample size are required to validate the effectiveness and safety of 577-nm semiconductive laser as a treatment modality for sub-ILM hemorrhage.

Sub-inner limiting membrane hemorrhage is seldom seen in clinical settings, and OCT is an effective tool for diagnosis. The 577-nm semiconductive laser is effective and safe for sub-ILM hemorrhage treatment. The ILM, fovea, and outer retina are three sites that should be closely followed for complications after laser membranotomy.

## Data Availability Statement

The original contributions presented in the study are included in the article/**Supplementary Material**. Further inquiries can be directed to the corresponding author.

## Ethics Statement

The studies involving human participants were reviewed and approved by Beijing Tongren hospital. Written informed consent to participate in this study was provided by the participants’ legal guardian/next of kin. Written informed consent was obtained from the individual(s), and minor(s)’ legal guardian/next of kin, for the publication of any potentially identifiable images or data included in this article.

## Author Contributions

YZ contributed the interpretation of data and drafting of the report. XP contributed to the study design and review. All authors contributed to the article and approved the submitted version.

## Conflict of Interest

The authors declare that the research was conducted in the absence of any commercial or financial relationships that could be construed as a potential conflict of interest.

## Publisher’s Note

All claims expressed in this article are solely those of the authors and do not necessarily represent those of their affiliated organizations, or those of the publisher, the editors and the reviewers. Any product that may be evaluated in this article, or claim that may be made by its manufacturer, is not guaranteed or endorsed by the publisher.
