# Supplementary material for: Efficiency and Complication of 577-nm Laser Membranotomy for the Treatment of Retinal Sub-Inner Limiting Membrane Hemorrhage
Source: Front Ophthalmol (Lausanne). 2022 Jul 13;2:935188. doi: 10.3389/fopht.2022.935188 (PMC11182147; doi:10.3389/fopht.2022.935188)
Supplement: Supplementary file 1 [file DataSheet_1.docx]

**The figures and medical history of 19 cases**


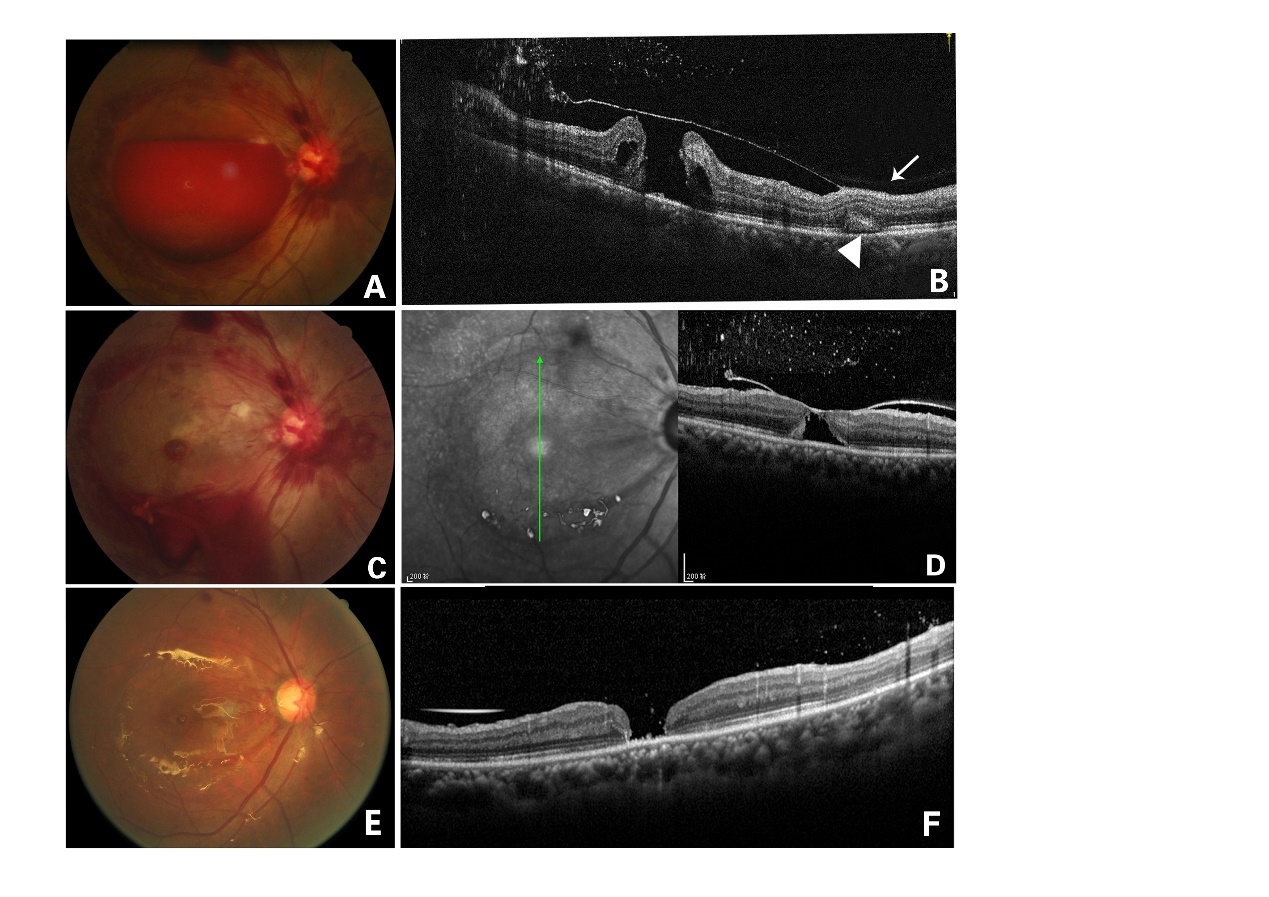


**Case 1.** Fundus photograph of the right eye showed a large sub-ILM hemorrhage **(A)**. OCT image of the right eye 4 days after laser showed ILM and ILM hole, multiple punctate hyperreflexia in the vitreous cavity. Full-thickness macular hole under the ILM could be seen. Posterior vitreous cortex was in front of the ILM attached to the retina（arrow）, “Patchy-like” structue located in the outer retina(arrowhead)**（B）.**The fundus photograph on the day of laser, blood flowed out from the hole of ILM, Vitreous bleeding and a round lesions of macula could be seen, which was later confirmed as macular hole**（C）**. OCT two weeks later, the ILM fell back and attached to the surface of the macular hole but the macular hole did not close**（D）**. Fundus photograph of right eye two months after laser treatment, vitrectomy with ILM peeling and silicone oil filling were performed in the right eye**（E）**. OCT two months later after vitrectomy showed that the macular hole was still not closed**（F）**.


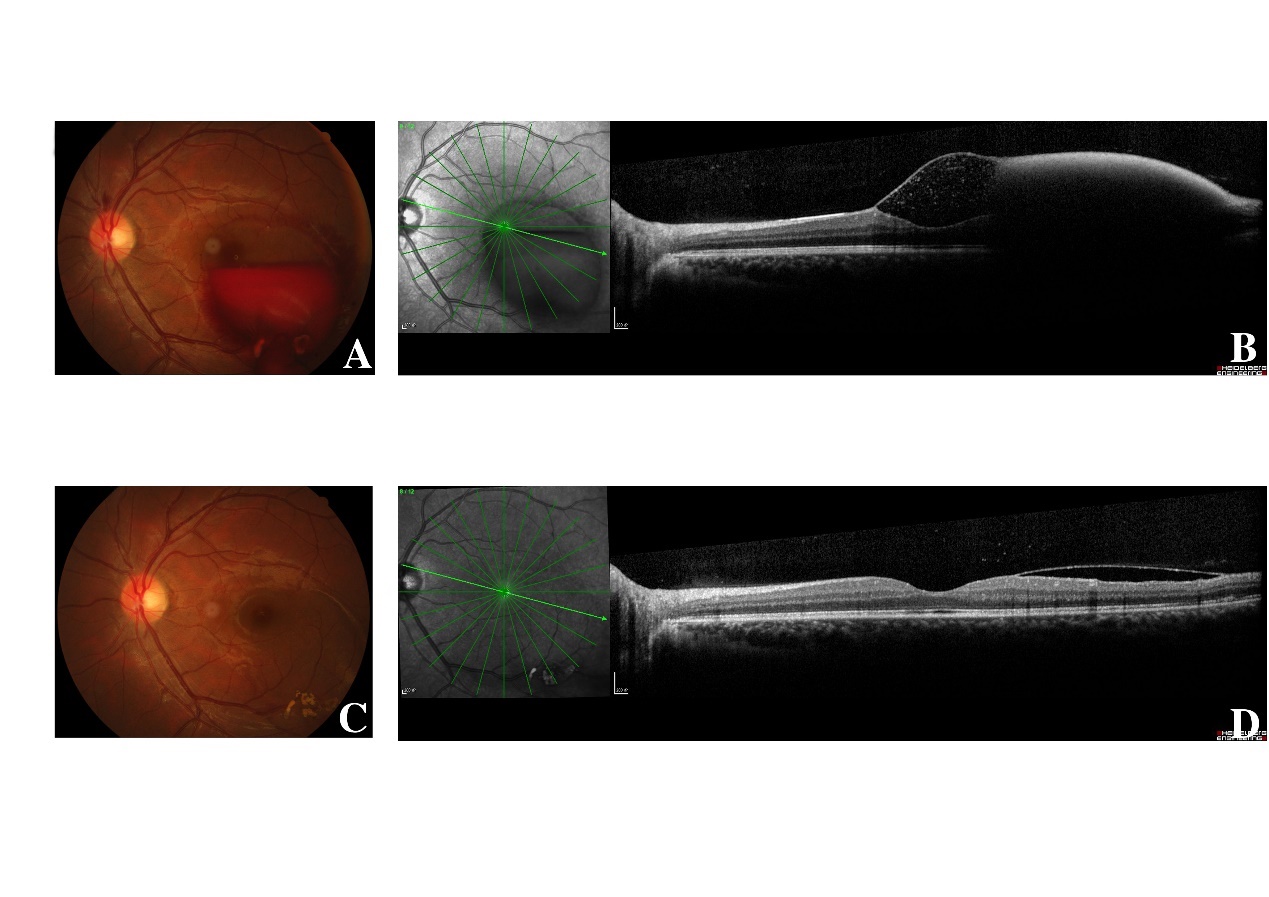


**Case 2.** Fundus photograph of the left eye showed sub-ILM hemorrhage on the day of laser and blood flowed out from the hole of ILM **(A)**. OCT of the left eye on the day of laser. ILM attached to the retina. In the lower part of sub-ILM cavity there was the level of blood with hyperreflexia. In the upper part there was multiple punctate hyperreflexia**（B）.**The fundus photograph 2 weeks after laser. The sub-ILM hemorrhage was absorbed completely in the macular region and the BCVA was recovered to 20/20**（C）.** OCT of the left eye 2 weeks after laser. The sub-ILM hemorrhage had been absorbed but the ILM did not r fall to the retina in the lower part of macula**（D）.**


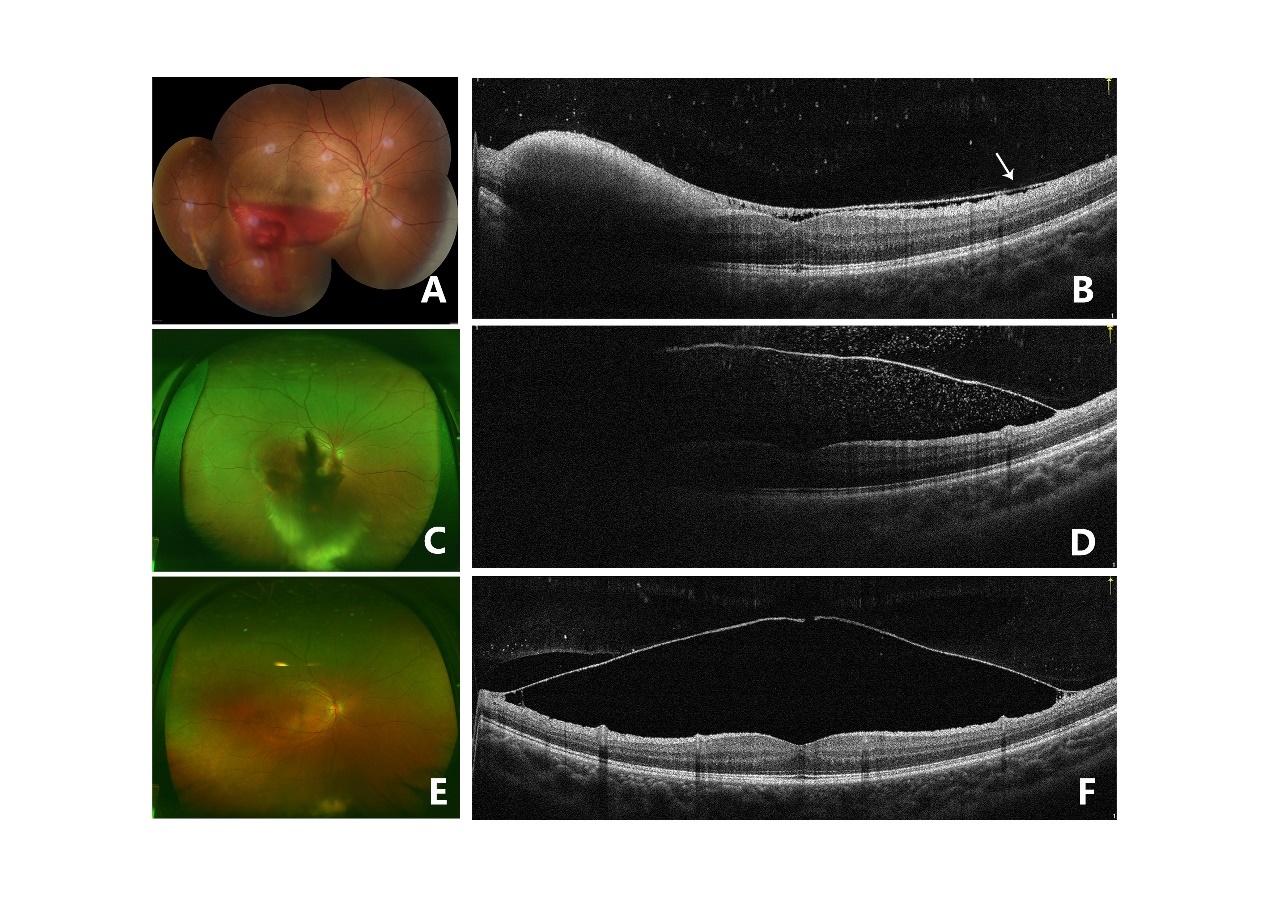


**Case 3**. Fundus photograph of the right eye showed sub-ILM hemorrhage 5 days after laser membranotomy. Blood could be seen flow out from the ILM hole **(A)**. OCT on the 5th day after laser treatment in the right eye showed that the ILM above the macula dropped and a thin hyporeflective band above the anterior surface of ILM, which was the posterior vitreous cortex (arrow). The hyperreflective spots could be seen on the retinal surface of the ILM, and residual hemorrhage under ILM could still be seen in the lower part of macular fovea. The contour of macula fovea was normal but the surface of retinal nerve fiber layer was undulant. Hyperreflective point could be seen in the vitreous cavity. The BCVA improved to 20/40 **(B)**. The fundus photograph one month later after laser treatment, the right eye developed sub-ILM hemorrhage and vitreous hemorrhage again, and the BCVA decreased to 20/400 **(C)**. OCT of the right eye one month after laser treatment showed that the ILM was stiff and protruding, and there were hyperreflective points inside and outside the ILM **(D)**. The fundus photography showed that the sub-ILM hemorrhage had been absorbed 9 months after laser, and vitreous opacity could be seen in the lower periphery of vitreous cavity **(E)**. OCT of the right eye 9 months after laser showed that the ILM hole did not heal and a cavity was formed below. Retinoschisis was seen at the attachment of ILM to retina **(F)**.


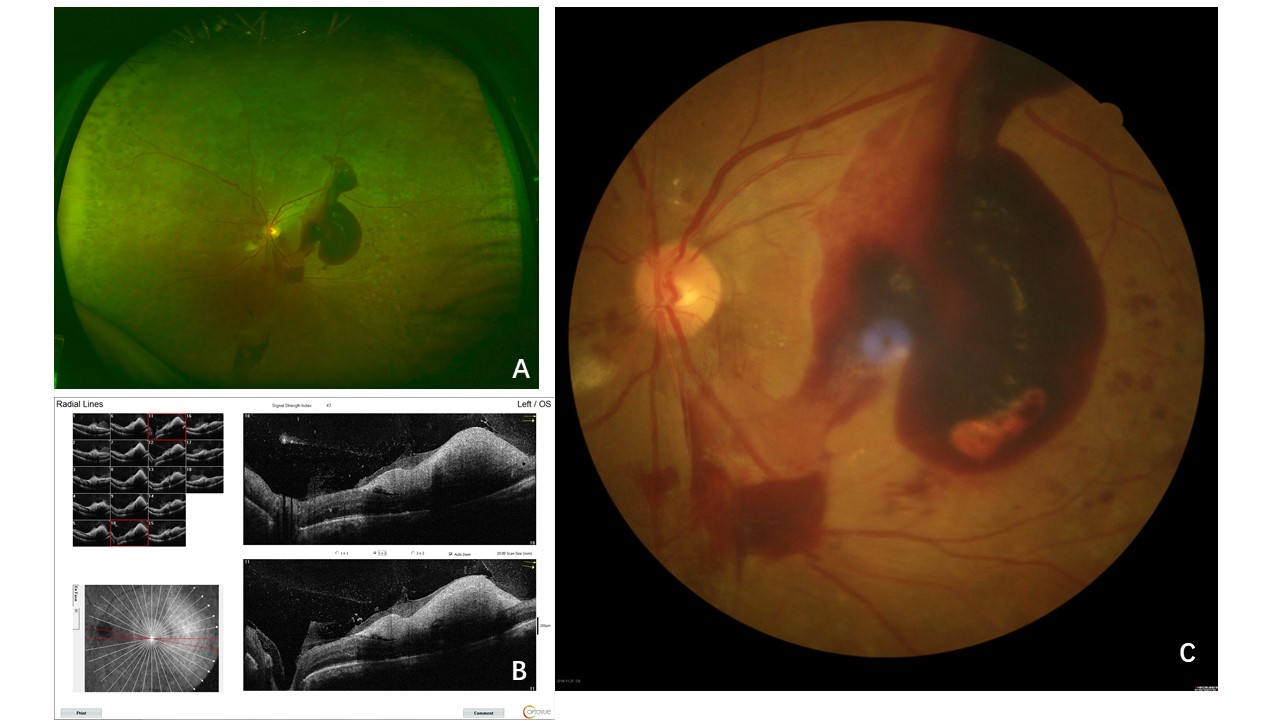


**Case 4**: Fundus photography of the left eye showed sub-ILM hemorrhage in the macula for 60 days of a patient with diabetic retinopathy **(A).** The OCT showed sub-ILM hyperreflexia **(B).** Fundus photography of left eye on the day of laser. The laser spots could be seen in the lower part of lesion but the sub-ILM hemorrhage drainage was unsuccessful **(C)**.


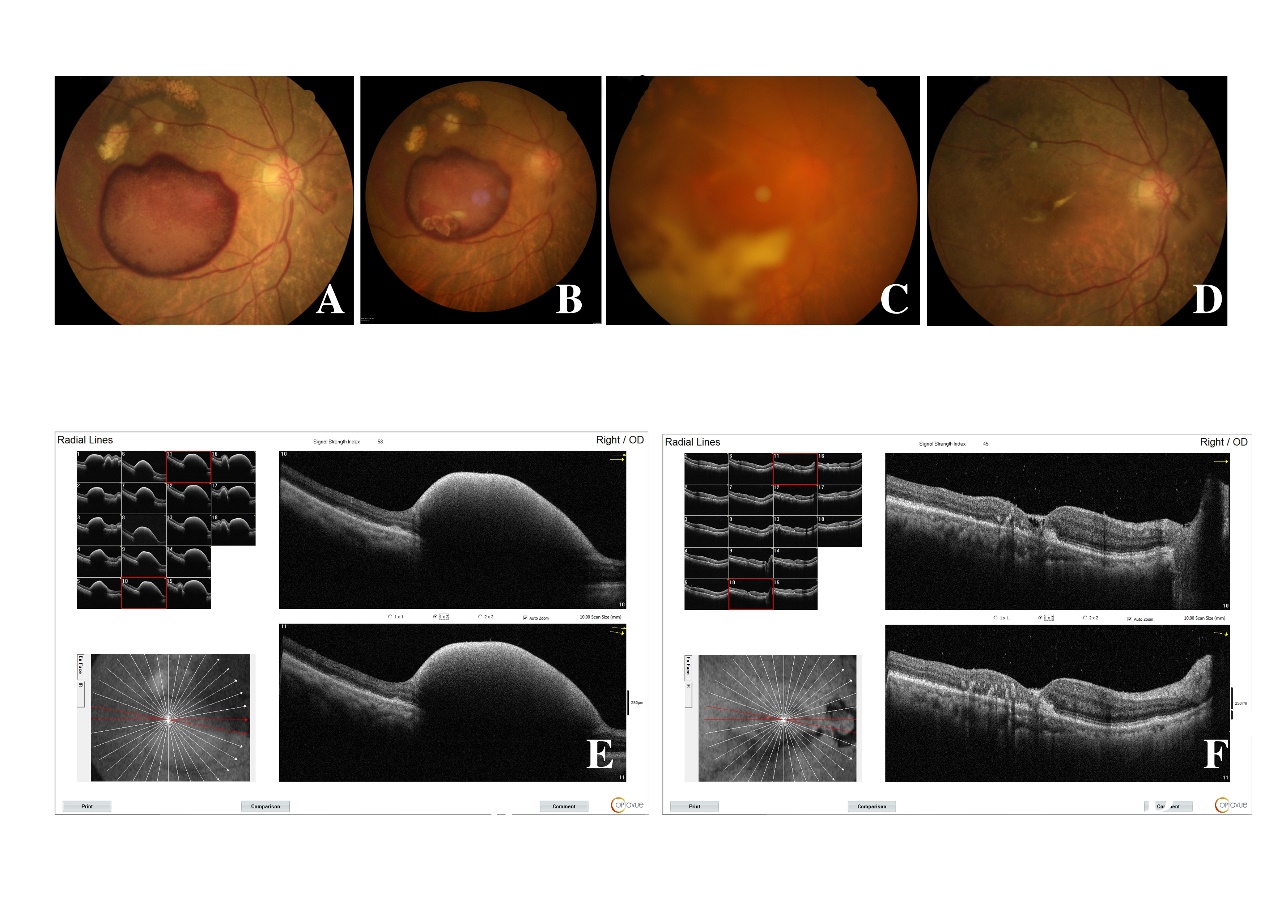


**Case 5.** Fundus photograph of the right eye showed sub-ILM hemorrhage in the macula one day before laser. In the upper vascular arch the retinal macroaneurysm cound be seen.**(A)**. Fundus photograph of the right eye on the day of laser. laser spots could been seen in the lower party of sub-ILM hemorrhage, but the blood was not drained immediately**(B)**. Fundus photograph of the right eye 1 week after laser**.** Blood was drained to the vitreous cavity **(C)**. Fundus photograph of the right eye 2 months after laser and the sub-ILM hemorrhage was absrobed completely**(D)**. OCT of the right eye 1 days before laser showed sub-ILM hemorrhage. There was”peg-like structure” in the outer retina **(E)**. OCT of the right eye 2 months after laser. The sub-ILM hemorrhage had been absorbed completely and the IIM attached to retina almost completely except the macular fovea. The “peg-like structure”in the outer retina had disappeared **(F)**.


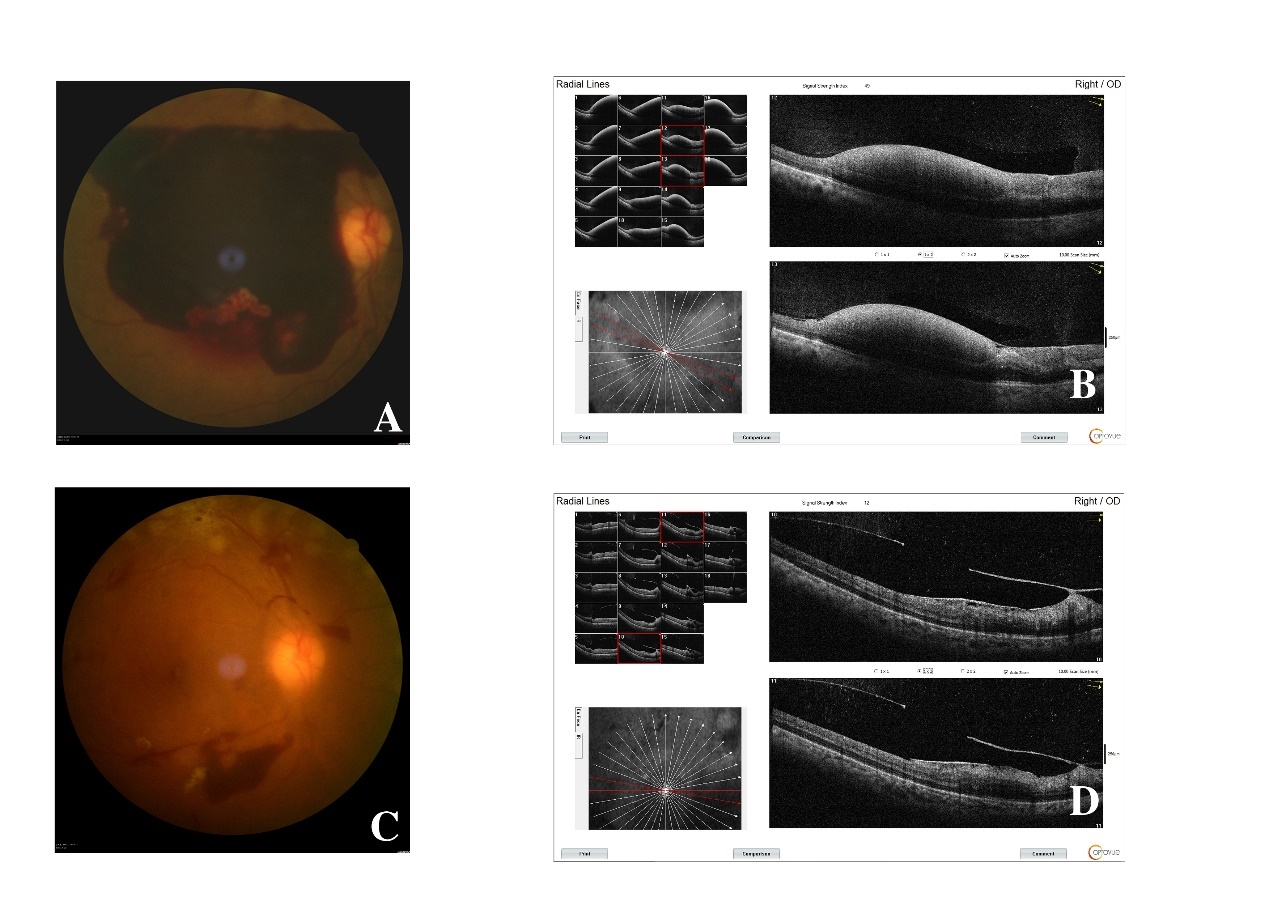


**Case 6.** Fundus photograph of the right eye showed sub-ILM hemorrhage with iregular shape in the macula on the day of laser **(A)**. OCT of the right eye on the day of laser showed sub-ILM hemorrhage with hyperreflectivity beneath the ILM**(B)**. Fundus photograph of the right eye 2 weeks after laser showed the sub-ILM hemorrhage had been absorbed in the region of macula. As this was a patient of proliferative diabetic retinopathy, so panretinal photocoagulation was performed **(C)**. OCT of the right eye 1 month after laser. The sub-ILM hemorrhage was absorbed completely but the ILM was not falling back to retina and the hole of he ILM could be seen. There was pre-macular membrane formation after laser **(D)**.


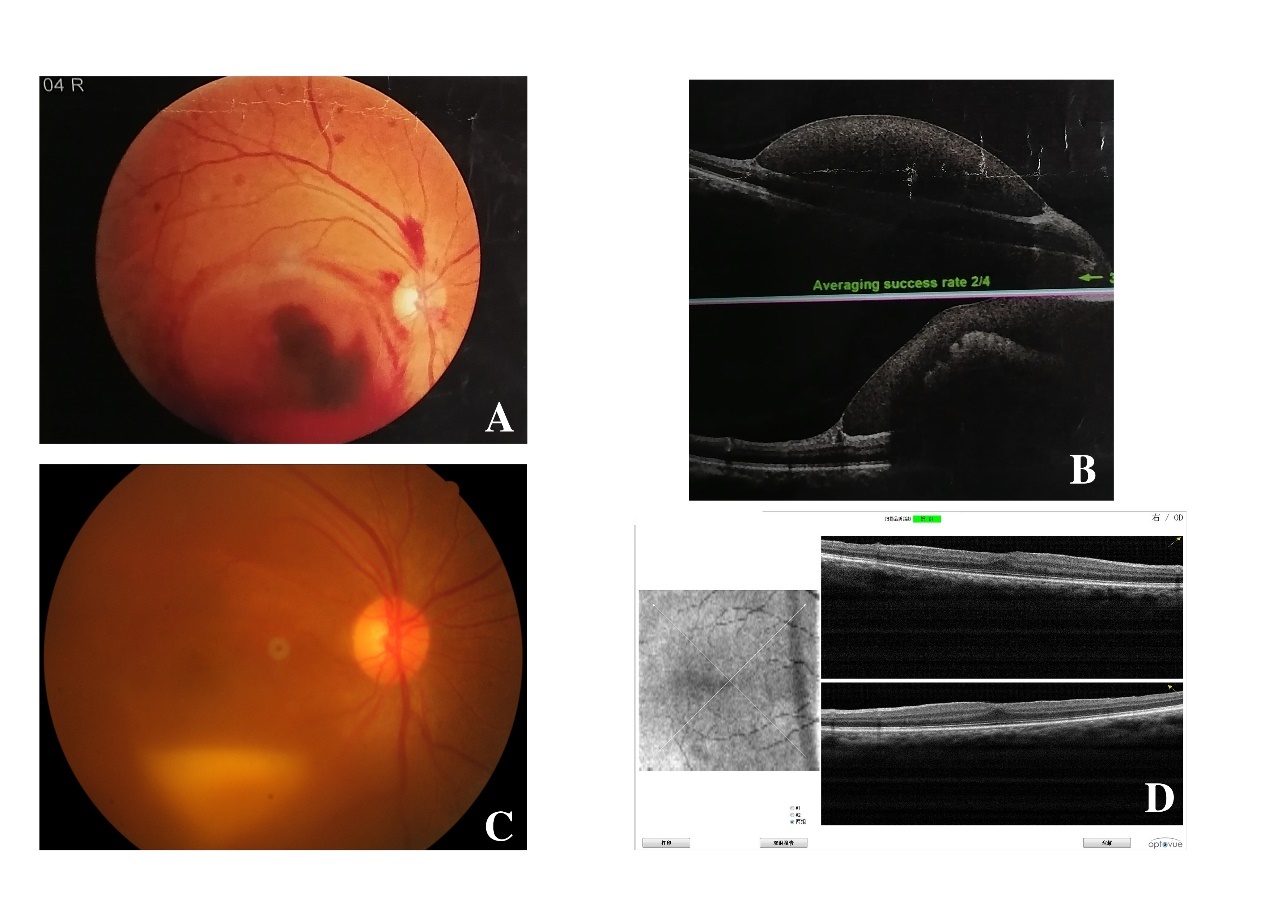


**Case 7.** Fundus photograph of the right eye showing sub-ILM hemorrhage before laser (The picture was taken from other hospital and was not clear) **(A)**.The OCT before laser showed the sub-ILM hemorrhage (The picture was taken from other hospital and was not clear) **(B)**. Fundus photograph 1 month after laser showed vitreous opacity and the patients received vitrectomy 2 months after laser**(C)**. The OCT 8 months after vitrectomy showed the normal contour of macula**(D)**.


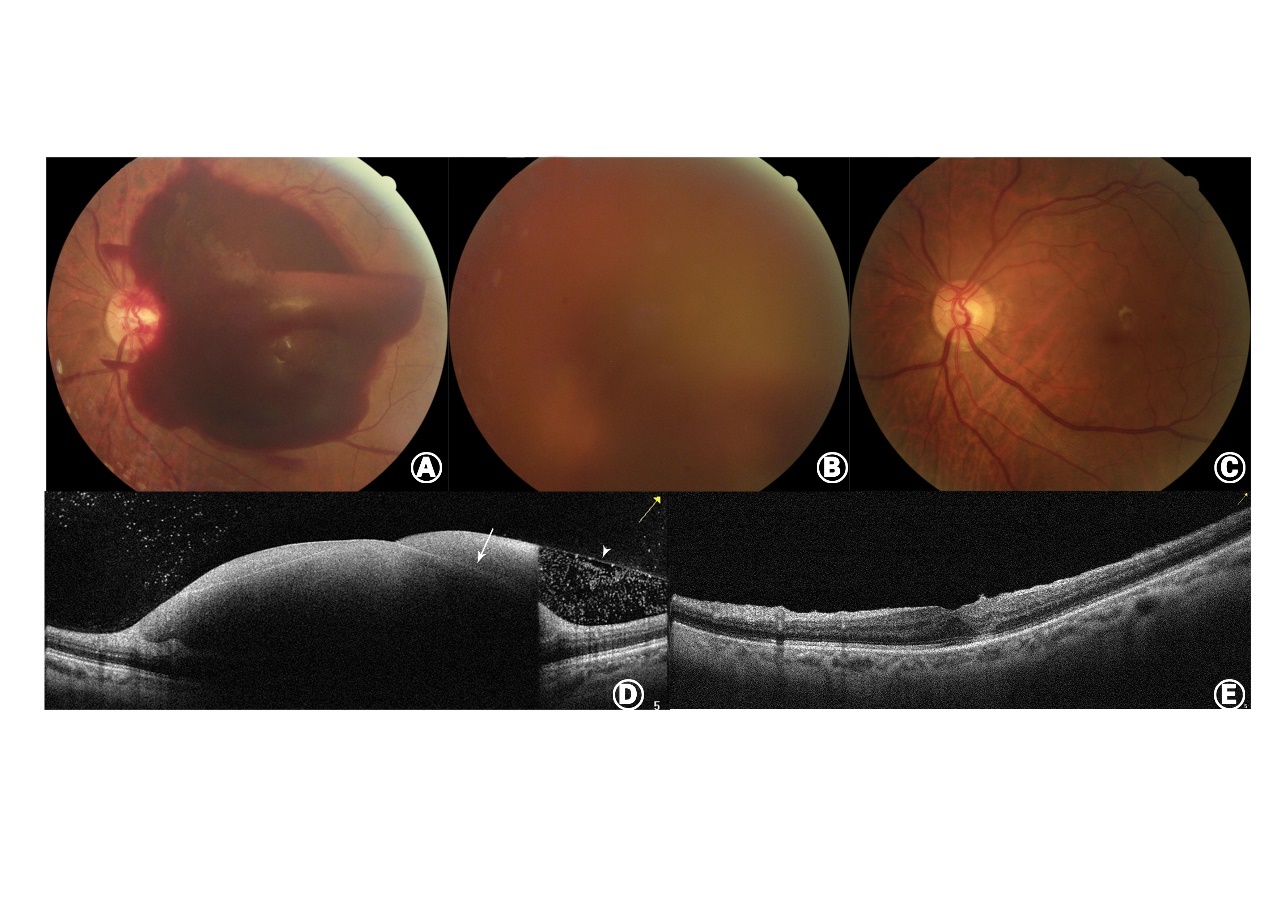


**Case 8** Fundus photograph of the left eye showed coexistence of sub-ILM hemorrhage (kidney shape) and subhyaloidal hemorrhage (with blood level)**.** In this case both hemorrhage had glistening light reflex **(A)**. Fundus photograph of the left eye after laser. The sub-ILM hemorrhage had been drained into the vitreous cavity but did not absorb for 3 months and the OCT could not be obtained. The patient received vitrectomy 3.5 months after laser **(B)**. Fundus photograph of the left eye 4 days after vitrectomy showed the vitreous bleeding had been cleaned and the BCVA recovered from finger count to 20/20 **(C)**. OCT of the left eye before laser showed the sub-ILM hemorrhage (arrow) and subhyaloidal hemorrhage (arrowhead) **(D)**. OCT of the left eye 4 days after vitrectomy with ILM peeling showed the almost normal contour of the macula fovea**(E)**.


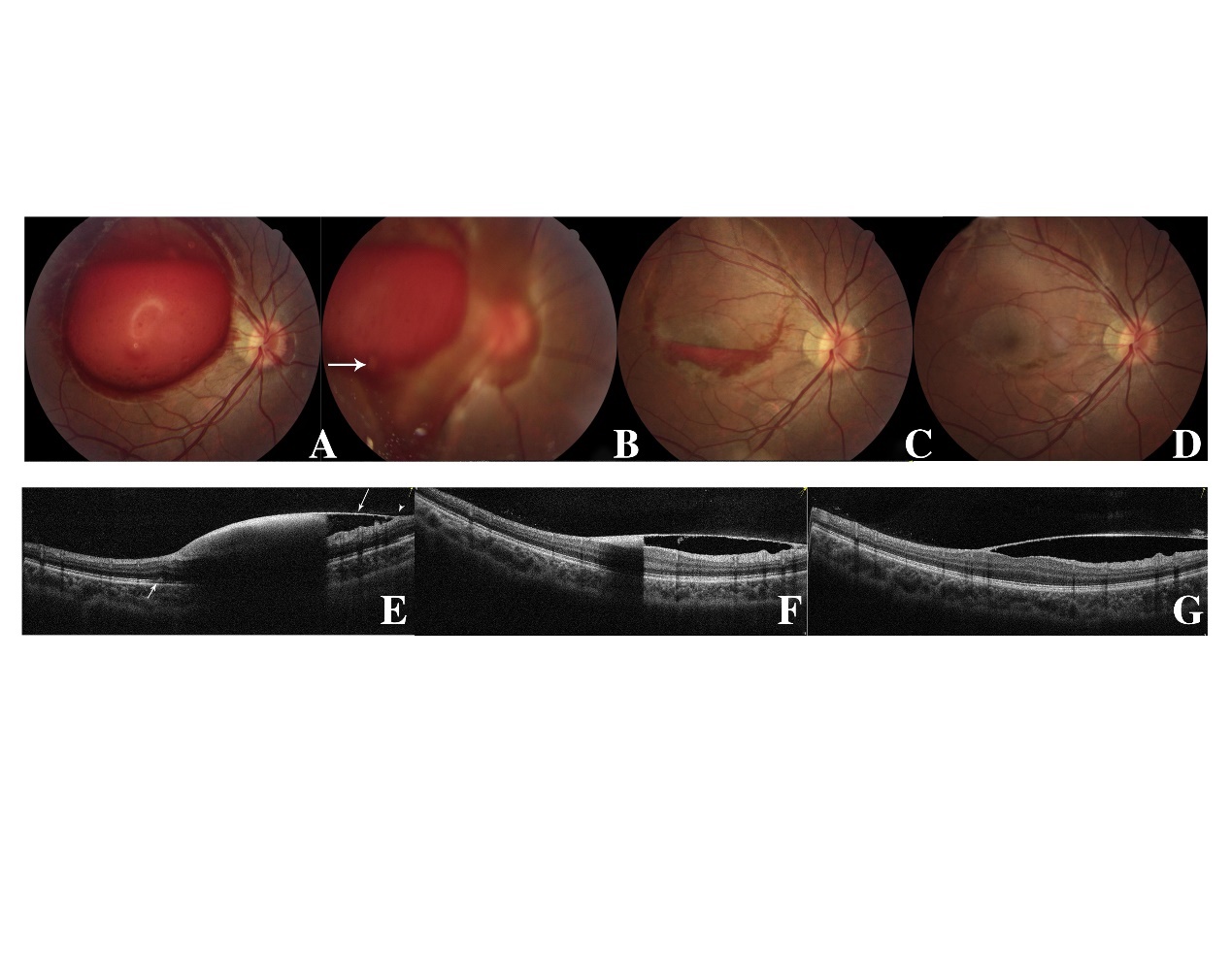


**Case 9**. Fundus photograph of the right eye showed sub-ILM hemorrhage with “Arcus retinalis” before laser **(A)**. Fundus photograph of the right eye on the day of laser showed hemorrhage was drained into the vitreous(arrow)**(B)**. Fundus photograph of the right eye 2.5 months later after laser showed the sub-ILM hemorrahge had been absorbed almostly but not completely **(C)**. Fundus photograph of right eye 7 months after laser. The hemorrhage was absorbed completely and the BCVA recovered from 20/1000 to 20/20**(D)**.The OCT showed sub-ILM hemorrhage before laser. The ILM (long arrow) and the posterior vitreous cortex (arrowhead) cound be differetiated. There was “peg-like structure” in the outer retina(short arrow)**(E)**. The OCT 2 months after laser showed the sub-ILM hemorrhage was not absorbed completely **(F)**. The OCT 7 months after laser showed the sub-ILM hemorrhage was absorbed completely but the ILM was rigid and not falling back to the retina, which formed “cavity-structure” beneath the ILM. The “peg-like structure” in the outer retina disappeared**(G)**.


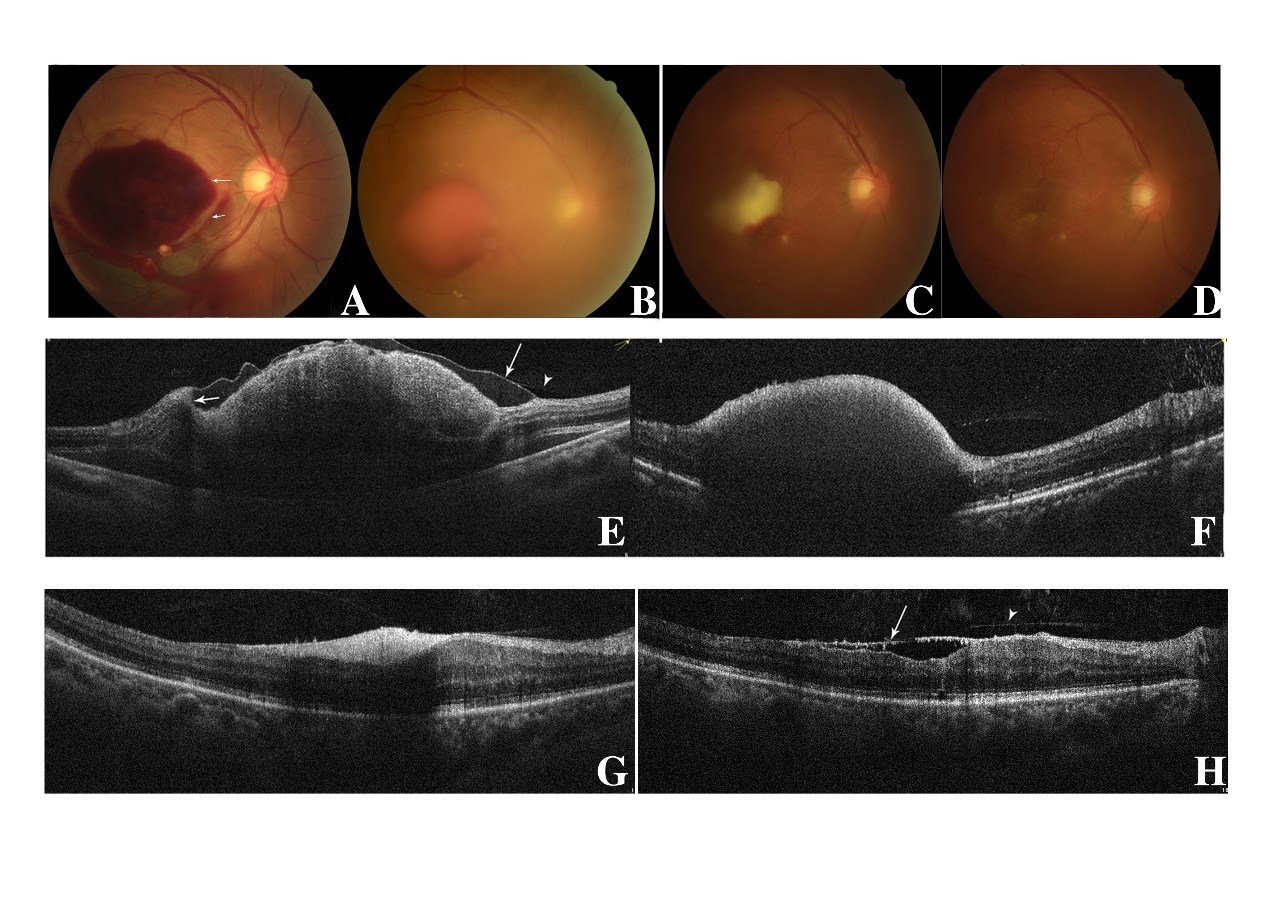


**Case 10**. Fundus photograph of the right eye showing sub-ILM hemorrhage on the day of laser membranotomy. Blood could be seen flow out from the ILM hole. There were two “ring” of hemorrhage which represented two kinds of sub-ILM hemorrhage as liquefying (short arrow) and coagulative (long arrow). This is different from “double ring” sign with the “inner ring” caused by the sub-ILM hemorrhage and the “outer ring” caused by the sub-hyaloid hemorrhage. In the lower part there was subretina hemorrhage **(A).** 1.5 months later the sub-ILM hemorrhage became smaller but had not been absorbed completely **(B)**. 3 months after laser the sub-ILM hemorrhage became even smaller but had not been absorbed completely. The color of sub-ILM hemorrhage changed from red to yellow-white **(C)**. 4 months after laser the sub-ILM hemorrhage had been absorbed completely and the BCVA recovered from 20/1000 to 20/50**(D)**.The OCT of right eye on the day of laser. The posterior vitreous cortex (arrowhead) and the wavy-like ILM (long arrow) could be differentiated. The blood level of sub-ILM hemorrhage could be seen (short arrow) **(E).** The OCT 1.5 months later after laser. the sub-ILM hemorrhage had not been absorbed completely but the ILM had fallen back to retina **(F).** The OCT 3 months later after laser. The sub-ILM hemorrhage had became more smaller and more dense but had not been absorbed completely**(G)**. The OCT 4 months after laser showed the sub-ILM hemorrhage had been absorbed completely and the IIM attached to retina almostly completely except the macular fovea (arrow showed the ILM and arrowhead showed the posterior vitreous cortex)**(H)**.


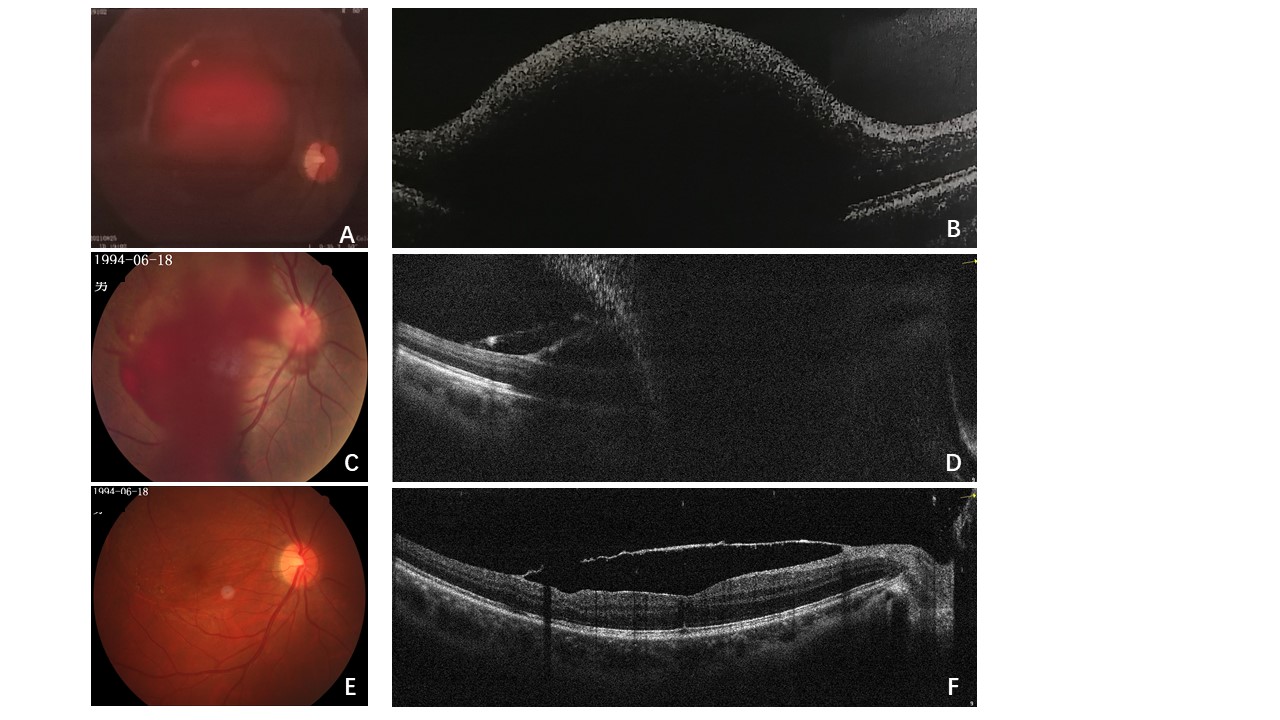


**Case 11**. Fundus photograph of the right eye showing sub-ILM hemorrhage before laser (The picture was taken from other hospital and was not clear) **(A).** The OCT before laser showed the sub-ILM hemorrhage (The picture was taken from other hospital and was not clear) **(B).** Fundus photograph of right eye on the day of laser. blood cound be seen flowed from the laser hole of ILM **(C).** OCT of right eye on the day of laser. The structure of macula could not been distinguished because of sub-ILM hemorrhage drainage into the vitreous body **(D)**. Fundus photograph of right eye 1 week after laser. The hemorrhage in the macula had disappeared and the BCVA had increased from 20/200 to 20/40 **(E).** The OCT of right eye 1 week after laser. the lase hole could be seen in the Lower part of ILM. The ILM is rigid and the sub-ILM cavity could be seen **(F).**


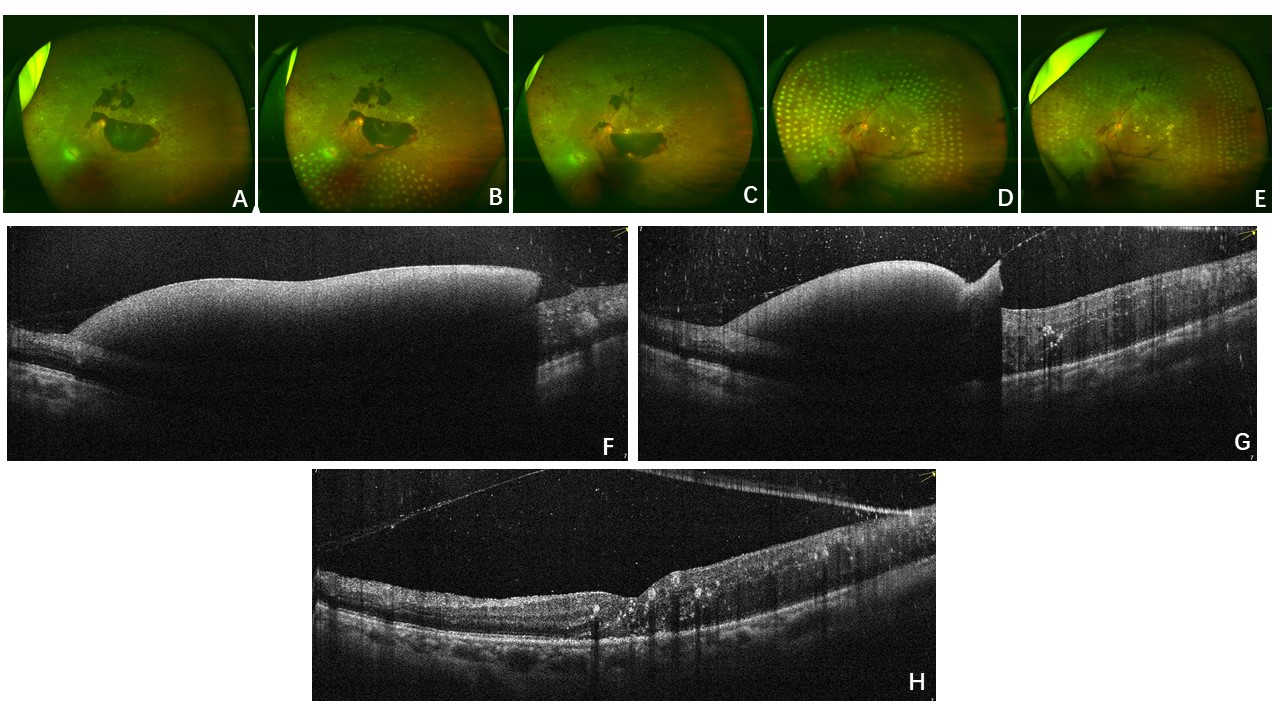


**Case 12.** Ultra-wide-field fundus photograph (Optos) of the left eye showed sub-ILM hemorrhage with blood level in the macula. The patient had history of DM for 15 years and hemorrhage and exudate could been seem in the retina **(A).** The fundus photograph of left eye on the day of laser. the laser spots could be seen in the lower part of the ILM but hemorrhage was not drained at that same time. The laser of lower part of retina was performed for PDR**（B）**. One week later the sub-ILM hemorrhage had been drained partly and the macular fovea could be seem. The BCVA of right eye changed from finger count to 20/400 **(C).** Three weeks after laser the macular region became visible and the sub-ILM hemorrahge was drained into the viterous cavity. PRP was continued **(D)**. 1 month later the PRP was completed **(E)**. The OCT before laser. There was dense hyperreflexia under the ILM **(F).** The OCT 1 week after laser. In the lower part of sub-ILM cavity there was the level of blood with hyperreflexia. In the upper part the blood disappeared **(G).**1 month after first laser, the sub-ILM hemorrhage disappeared totally, but the ILM was not falling back and sub-ILM cavity was formed **(H).**


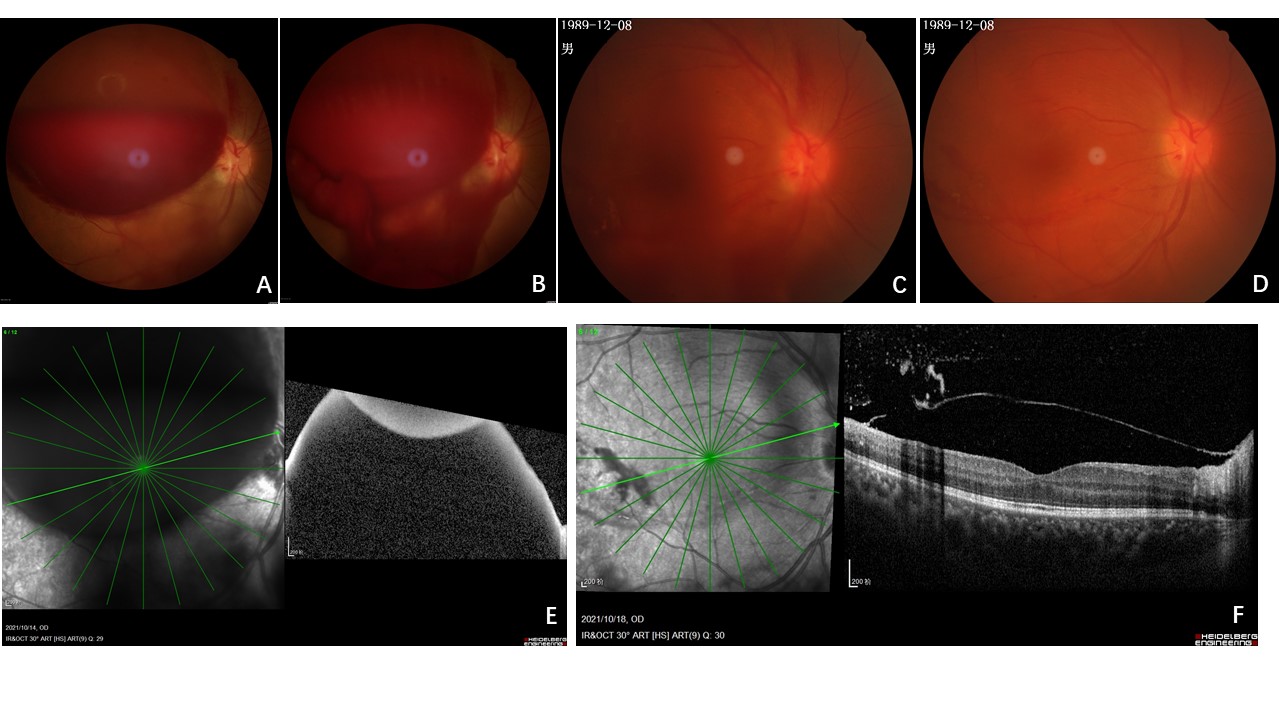
**Case 13**. The fundus photograph of the right eye showing sub-ILM hemorrhage before laser **(A)**. The fundus photograph of the right eye on the day of laser. Blood could be seen flowing from the laser hole on the ILM **(B).** The fundus photography of the right eye the first day after laser. The sub-ILM hemorrhage had been drained almost completely **(C)**. The fundus photography of the right eye 4 days after laser. The sub-ILM hemorrhage had been drained completely and the BCVA changed from finger count to 20/125 **(D)**. The OCT before laser of right eye showed the hyperreflexia under ILM **(E)**. The OCT 4 days after laser showed the sub-ILM hemorrhage had been drained completely. The laser hole on the ILM could be seen and the macular center contour was normal **(F).**


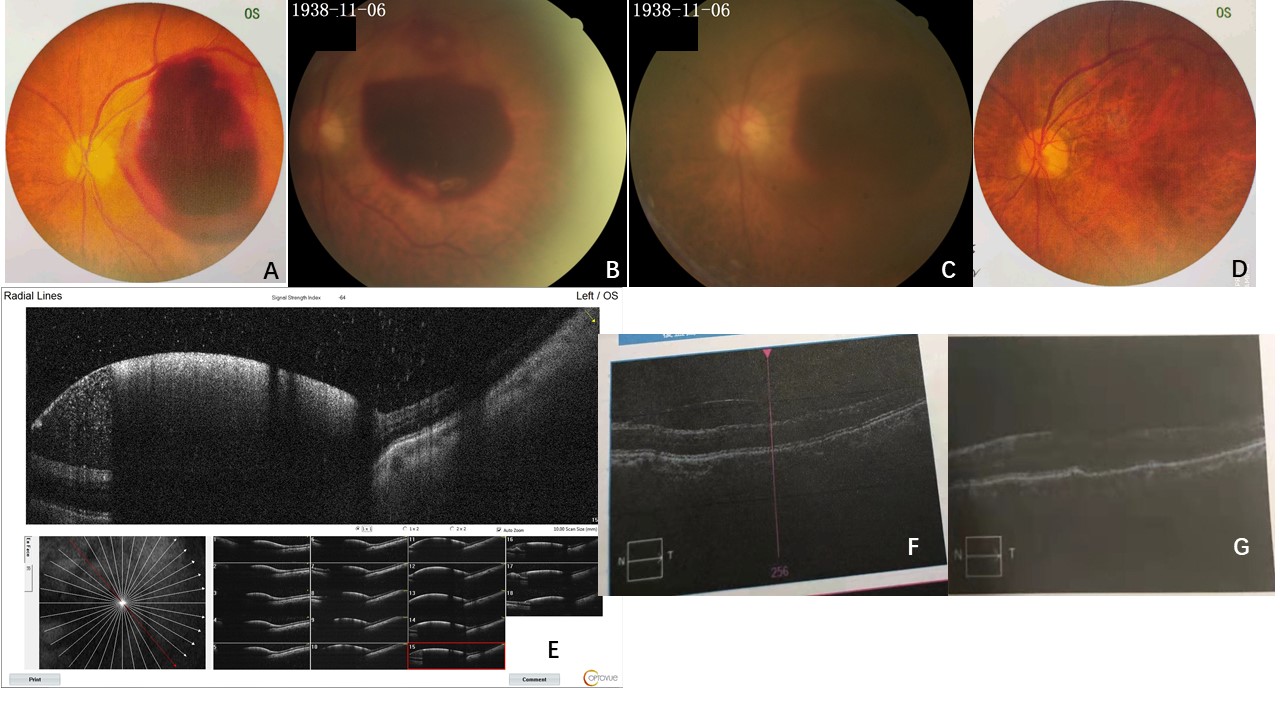


**Case 14.** The fundus photograph of the left eye showing sub-ILM hemorrhage before laser treatment (The picture was taken from other hospital and was not clear) **(A).**The day on the laser the photo showed the laser spots on the surface of inner limiting membrane but the hemorrhage had not been drained immediately**(B).** Six days after laser the sub-ILM hemorrahge had been drained gradually**(C).** Three months later the hemorrhage had been absorbed completely (The picture was taken from other hospital and was not clear)**( (D).**The OCT showed sub-ILM hemorrhage**(E).** Three months later the OCT showed sub-ILM hemorrhage had been absorbed and ILM had followed down to the retina (The pictures were taken from other hospital and was not clear)**( (F) and (G).**


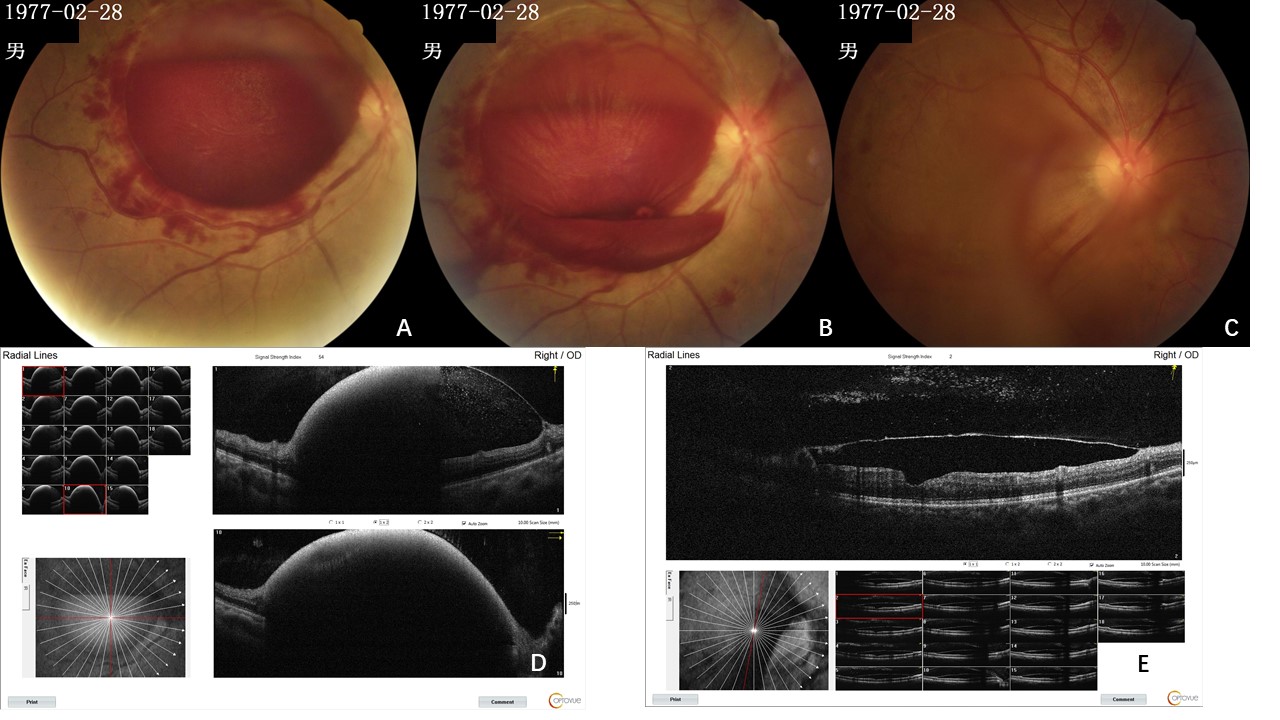


**Case 15.** The photography showed the sub-ILM hemorrhage with retinal hemorrhage **(A).** the day on the laser showed the sub-ILM hemorrhage had been drained immediately after laser into the pre-retina space **(B).** Two weeks later the hemorrahge had diffused into the vitreous body**(C).** The OCT before laser showed the sub-ILM hemorrhage with liquid level**(D).** Two weeks later the sub-ILM hemorrhage had been drained completely and the sub-ILM cavity was formated**(E).**


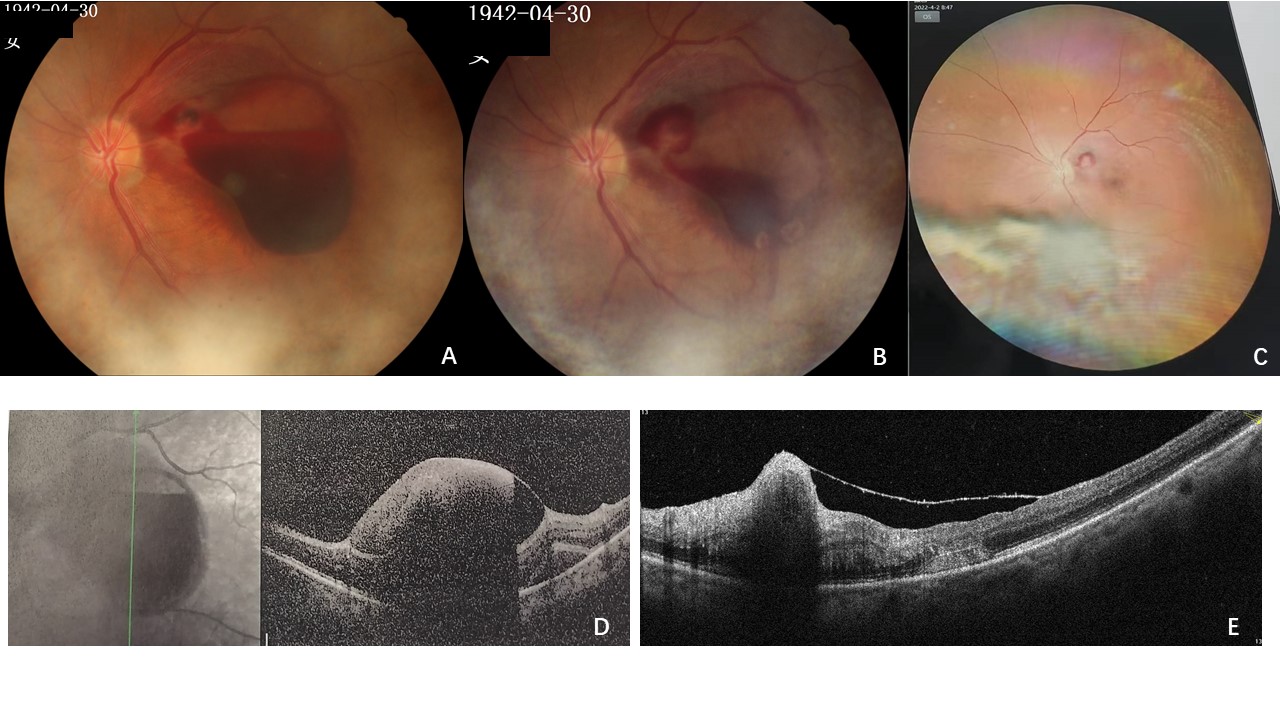


**Case 16** The photography showed the sub-ILM hemorrhage with retinal hemorrhage**(A).** the day on the laser showed the sub-ILM hemorrhage had been drained immediately after laser into the pre-retina space **(B).** One month later, the vitreous opacity was still obvious but the sub-ILM hemorrhage had been absrobed completely**(C).**The OCT before laser, the sub-ILM was predominent and the “pathy-like” structure cound be seen in outer retina **(D).** The OCT after laser, the ILM was falling down but did not re-attach to the retina**(E).**


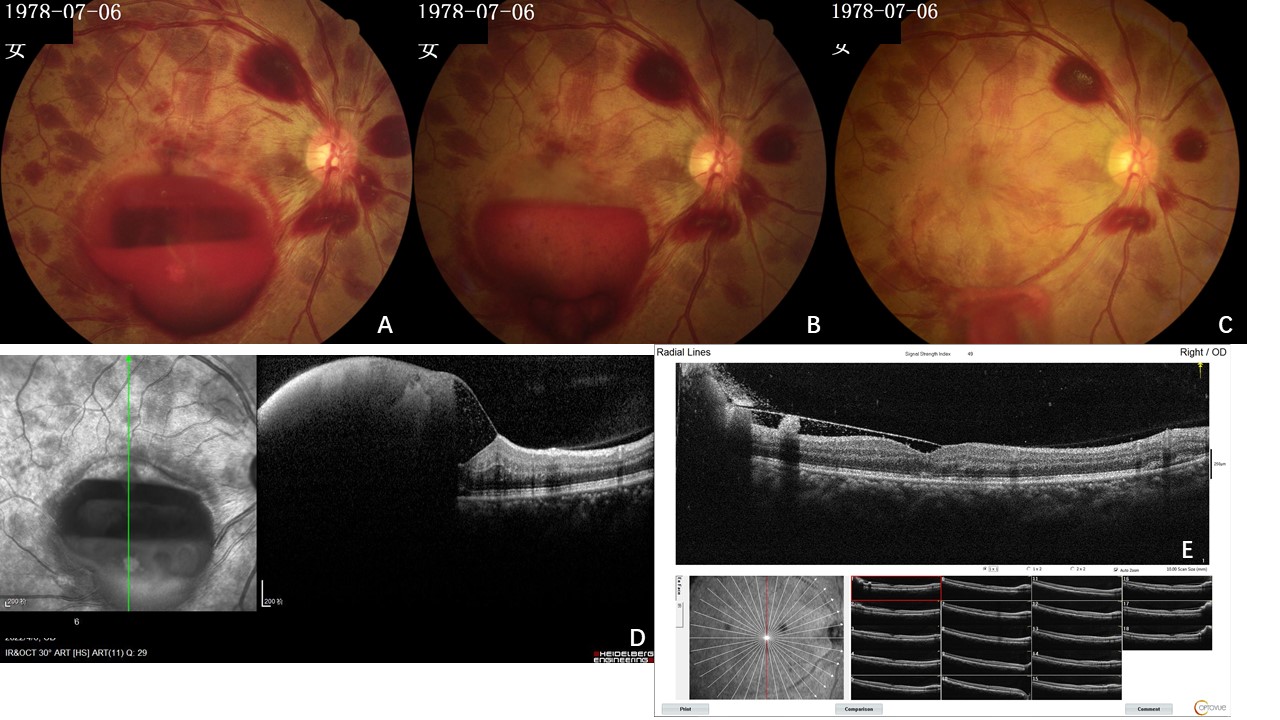


**Case 17** The photography showed the sub-ILM hemorrhage with retinal hemorrhage**(A).** the day on the laser showed the sub-ILM hemorrhage had been drained immediately after laser into the pre-retina space **(B).** 6 days after laser the sub-ILM hemorrhage had been drained almost completely**(C).** The OCT before laser the sub-ILM hemorrhage could be seen**(D).** The OCT after laser, the ILM was falling down but did not re-attach to the retina **(E)**


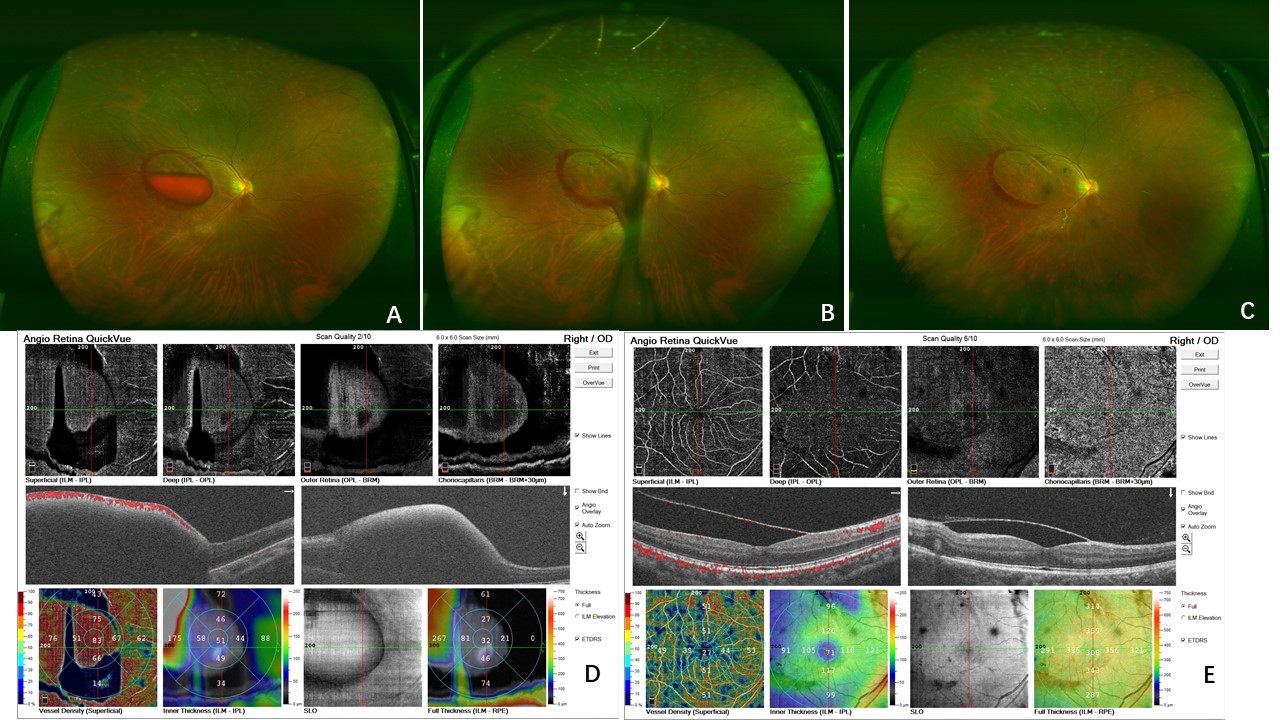


**Case 18.** The photography showed the sub-ILM hemorrhage with blood level **(A).** the day on the laser showed the sub-ILM hemorrhage had been drained immediately and the macular region was clear in 30 minutes**(B).** 5 days after laser the sub-ILM hemorrhage the BCVA recovered to 20/25**(C).** The OCTA before laser the sub-ILM hemorrhage could be seen**(D).** The OCTA after laser, the ILM was stiff and the sub-ILM cavity could be seen**(E)**


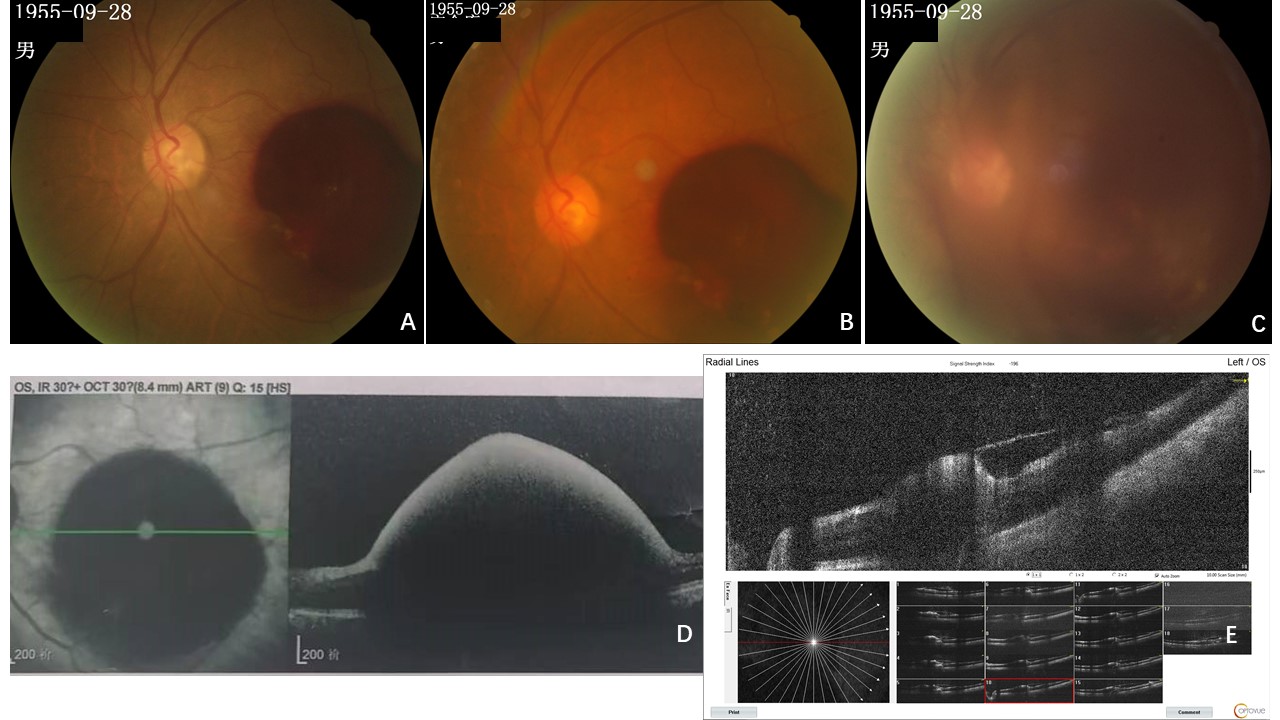


**Case 19.** The photography showed the sub-ILM hemorrhage without blood level**(A).** the day on the laser showed the sub-ILM hemorrhage had been drained immediately. Because the patient was deaf and the eye movement, the laser was not suffient and only one laser spot was effective **(B).** 5 days after laser the sub-ILM hemorrhage was drained into the vitreous body**(C).** The OCT before laser the sub-ILM hemorrhage could be seen**(D).** The OCT after laser, the ILM was falling down but did not re-attach to the retina **(E).**
